# Bispecific targeting of CHI3L1 and PD-1 as a therapeutic strategy for pulmonary fibrosis

**DOI:** 10.1172/jci.insight.201609

**Published:** 2026-04-28

**Authors:** Han-Seok Jeong, Takayuki Sadanaga, Joyce H. Lee, Suchitra Kamle, Bing Ma, Yang Zhou, Sung Jae Shin, Jack A. Elias, Chun Geun Lee

**Affiliations:** 1 Molecular Microbiology and Immunology, Brown University, Providence, Rhode Island, USA.; 2 Johns Hopkins University School of Medicine, Baltimore, Maryland, USA.; 3 Department of Microbiology, Institute for Immunology and Immunological Disease, Graduate School of Medical Science, Brain Korea 21 Project, Yonsei University College of Medicine, Seoul, Korea.; 4 Department of Medicine, Brown University, Providence, Rhode Island, USA.; 5 Intercollege, Hanyang University, Korea.

**Keywords:** Immunology, Pulmonology, Fibrosis, Macrophages, Molecular biology

## Abstract

CHI3L1, a chitinase-like protein, is implicated in pulmonary fibrosis, yet its mechanisms are incompletely understood. We demonstrated that CHI3L1 coordinates profibrotic macrophage activation and invasive myofibroblast differentiation, and their crosstalk. In vitro, CHI3L1 drove M2-like macrophage polarization with increased CD163, CD206, and PD-L1, and amplified TGF-β_1_–induced fibroblast responses, including myofibroblast transformation, migration, and invasion. Mechanistically, CHI3L1 enhanced TGF-β_1_ signaling through SMAD, AKT, and ERK pathways, and PD-L1 was required for CHI3L1/TGF-β_1_–driven myofibroblast transformation. Coculture studies further demonstrated the ability of CHI3L1 to induce profibrotic macrophage activation that enhanced myofibroblast transformation mediated via a CD44/PD-L1 axis. In vivo, following bleomycin challenge, CHI3L1-transgenic mice exhibited increased PD-L1^+^ M2 macrophages, PD-L1^+^PDGFRα^+^ fibroblasts, and PD-1^+^ immune cells compared with WT controls. Therapeutically, combined anti-CHI3L1 and anti-PD-1 antibodies, or a bispecific anti-CHI3L1–anti-PD-1 antibody, produced greater antifibrotic efficacy than monotherapy. These findings demonstrate crosstalk between CHI3L1 and the PD-1/PD-L1 pathway that promotes profibrotic macrophage activation and invasive fibroblast differentiation and support dual targeting of CHI3L1 and PD-1/PD-L1 as a promising therapeutic strategy for pulmonary fibrosis.

## Abstract 2

CHI3L1 promotes profibrotic macrophage activation and fibroblast interaction via the PD-1/PD-L1 axis, suggesting a therapeutic target in pulmonary fibrosis

## Introduction

Pulmonary fibrosis (PF) is a progressive and often fatal lung disease characterized by excessive deposition of extracellular matrix (ECM) proteins, leading to scarring and impaired lung function (1). Idiopathic pulmonary fibrosis (IPF) is the most common form of PF and has a median survival of only 2–3 years after diagnosis (2, 3). The pathogenesis of PF involves complex interactions among multiple cell types, including fibroblasts, myofibroblasts, and immune cells such as macrophages (4, 5). These cellular interactions establish a self-sustaining fibrotic cycle, marked by the activation and differentiation of macrophages and fibroblasts, which perpetuate fibrotic tissue remodeling (6, 7). Recent research has highlighted chitinase-3–like protein 1 (CHI3L1) as a crucial regulator in this setting (8, 9). However, the precise mechanisms through which CHI3L1 drives fibrosis remain incompletely understood.

The glycosyl hydrolase gene family 18 (GH18) contains chitinases and chitinase-like proteins (CLPs) that lack enzyme activity. CHI3L1 (also known as YKL-40; encoded by the *Chil1* gene in mice), the prototypic CLP, has been implicated in multiple biologic processes, including cell proliferation, migration, inflammation, and tissue remodeling associated with various diseases in the lung and other organs (10–14). Elevated levels of CHI3L1 have been observed in patients with IPF, which correlate with disease severity. These studies suggest that CHI3L1 contributes to PF by modulating immune responses and/or fibroblast activation (8, 9, 15). However, the mechanisms by which CHI3L1 promotes fibrotic pathways remain incompletely understood, particularly in the context of macrophage activation and myofibroblast differentiation.

Macrophages have been recognized as central players in the fibrotic response, with plasticity to adopt proinflammatory (M1) or antiinflammatory and profibrotic (M2) phenotypes in response to environmental stimuli (16, 17). The M2 phenotype is characterized by the expression of markers such as CD163 and CD206 and supports tissue repair and fibrosis through the secretion of growth factors and cytokines that drive fibroblast proliferation and activation (18, 19). Studies from our group and others have shown that CHI3L1 promotes the differentiation of monocytes into M2-like macrophages (15, 20), thereby contributing to the profibrotic milieu. In addition, we have found that CHI3L1 is a robust inducer of PD-L1 (21), an immune checkpoint molecule associated with immune evasion and chronic inflammation (22, 23). Surprisingly, the mechanisms underlying these responses have not been fully defined.

The role of the PD-1/PD-L1 axis in fibrotic diseases has gained interest, as it facilitates immune tolerance and promotes profibrotic responses (24, 25). In the context of PF, PD-L1 is implicated in the development of invasive fibroblasts responsible for persistent and progressive PF (26). Although CHI3L1 robustly induces PD-1/PD-L1 expression in immune and structural cells, including macrophages, fibroblasts, and epithelial cells in the tumor microenvironment, the mechanistic details of CHI3L1-driven macrophage differentiation and fibroblast activation and their coordination with the regulated expression of PD-1/PD-L1 require further investigation.

In this study, we investigated the roles of CHI3L1 in regulating profibrotic macrophage activation and invasive myofibroblast differentiation in the pathogenesis of PF. Through in vitro and in vivo experiments, we explored the interactions between CHI3L1, CD44, PD-L1, and key profibrotic pathways, including TGF-β signaling. Additionally, we assessed the therapeutic potential of bispecific targeting of CHI3L1 and the PD-1/PD-L1 axis in a murine model of bleomycin-induced PF. Our findings reveal mechanistic insights into the pathogenesis of PF and highlight the therapeutic promise of dual targeting of CHI3L1 and the PD-1/PD-L1 axis.

## Results

### IL-13 and TGF-β_1_ stimulate profibrotic macrophage activation via a CHI3L1-dependent mechanism.

**Figure 1:**
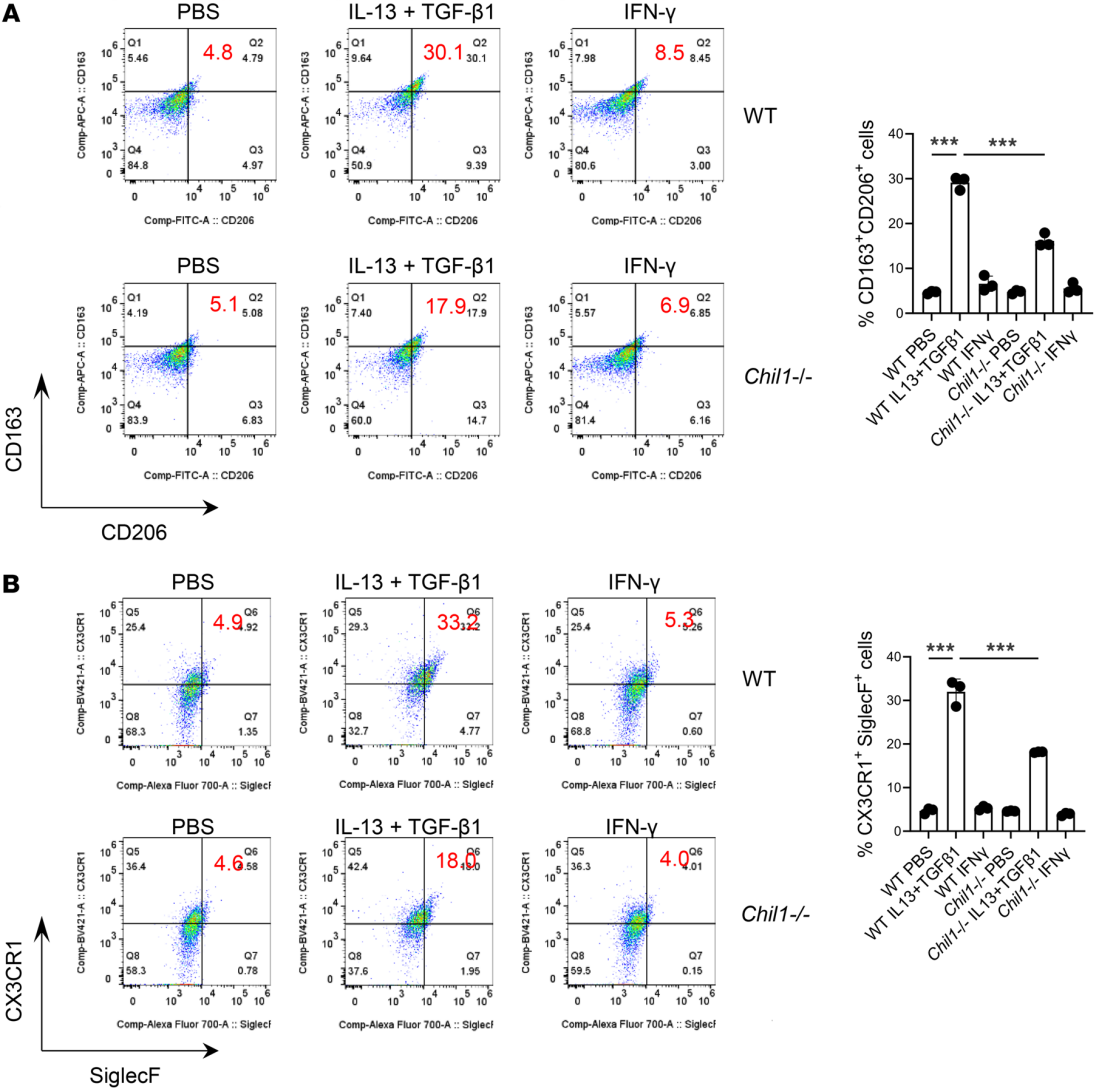
IL-13 and TGF-β_1_ stimulate M2 macrophage polarization via a CHI3L1-dependent mechanism. Bone marrow–derived macrophages (BMDMs) were isolated from WT and CHI3L1-null (*Chil1*^–/–^) mice and stimulated with recombinant IL-13 (20 ng/mL), TGF-β_1_ (5 ng/mL), and IFN-γ (20 ng/mL) for 72 hours. Cells were then analyzed by flow cytometry. (**A**) Frequency of CD206^+^CD163^+^ M2 macrophages. (**B**) Frequency of CX3CR1^+^SiglecF^+^ profibrotic macrophages. The bar graphs show the mean ± SEM from biological replicates. ***P < 0.001 by 1-way ANOVA followed by Šídák’s multiple-comparison test.

To investigate the role of CHI3L1 in macrophage profibrotic M2 polarization, we prepared bone marrow–derived macrophages (BMDMs) from WT and CHI3L1-null (*Chil1*^–/–^) mice and stimulated them with typical pro-M2 cytokines recombinant IL-13 and TGF-β_1_, with the pro-M1 cytokine IFN-γ as a negative control. After stimulation, we compared M2 and profibrotic macrophage populations by fluorescence-activated cell sorting (FACS) analysis. In WT BMDMs, IL-13 and TGF-β_1_ stimulation alone or together increased the frequency of CD163^+^ or CD206^+^ macrophages (Figure 1A and Supplemental Figure 1A; supplemental material available online with this article; https://doi.org/10.1172/jci.insight.201609DS1), whereas these increases were significantly reduced in CHI3L1-null BMDMs. Conversely, IFN-γ stimulation did not appreciably increase the frequency of CD163^+^ and CD206^+^ macrophages, and its effects were not significantly altered by loss of CHI3L1. Similarly, profibrotic CX3CR1^+^SiglecF^+^ macrophages were increased when treated with IL-13 and TGF-β_1_, but not with IFN-γ, and these increases were blunted in CHI3L1-null BMDMs (Figure 1B and Supplemental Figure 1B). These findings indicate that CHI3L1 is required for IL-13/TGF-β_1_–driven profibrotic macrophage polarization.

### Recombinant CHI3L1 induces profibrotic M2 macrophage differentiation and PD-L1 expression.

**Figure 2:**
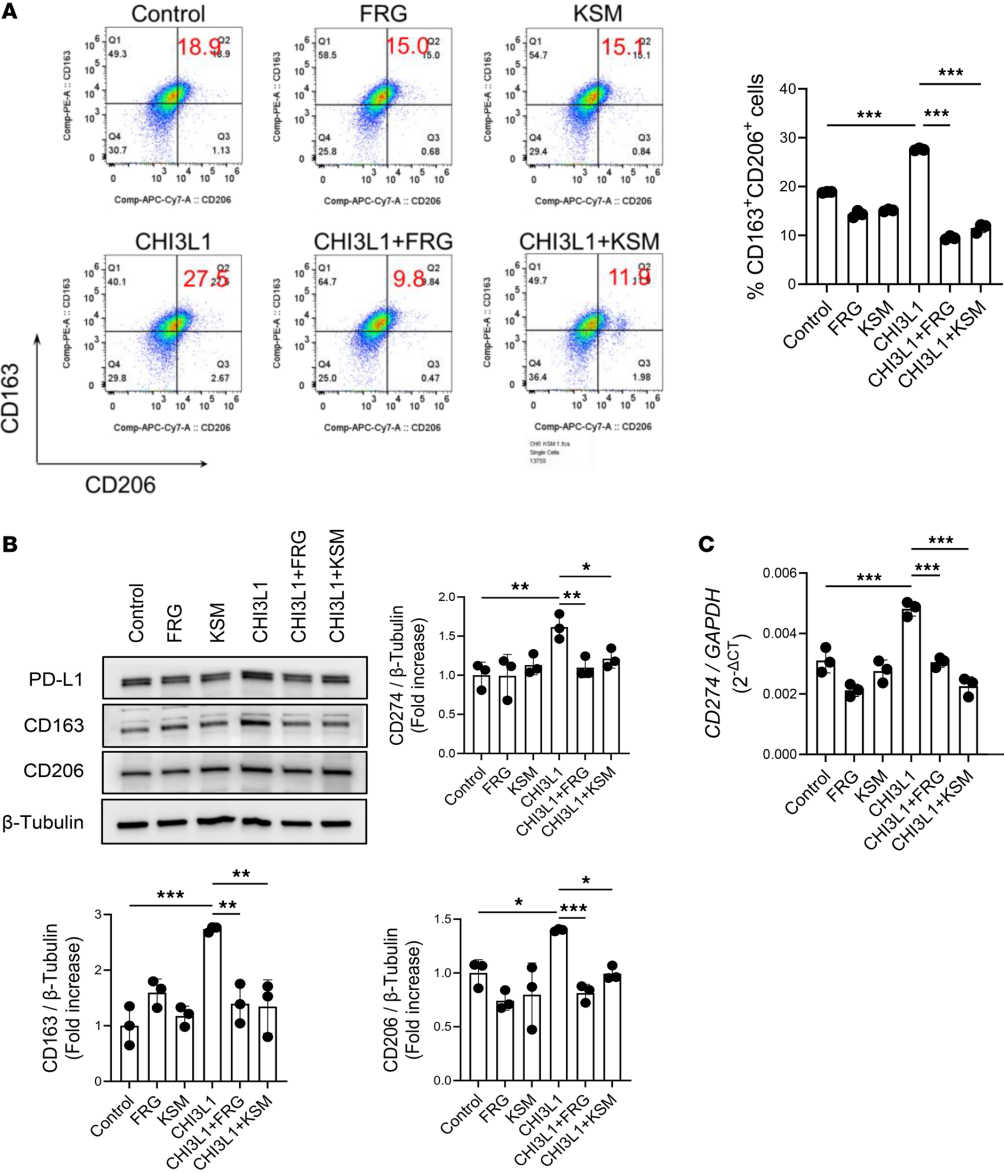
CHI3L1 drives M2 macrophage differentiation and PD-L1 expression. Human monocyte THP-1 cells were differentiated with PMA (100 ng/mL) for 24 hours, followed by 24 hours of rest and then stimulated with recombinant CHI3L1 (500 ng/mL) with or without anti-CHI3L1 antibody (FRG, 250 ng/mL) or kasugamycin (KSM, 250 ng/mL) for 24 hours. Expression of PD-L1, CD206, and CD163 was then evaluated. (**A**) Flow cytometry of the CD206^+^CD163^+^ M2 macrophages. (**B**) Western blot analysis of CD163, CD206, and PD-L1 protein expression. β-Tubulin was used as a loading control. (**C**) *CD274* (PD-L1) mRNA expression measured by quantitative RT-PCR. The bar graphs in **B** represent densitometric quantification of Western blot signals; values in **A**–**C** represent the mean ± SEM. *P < 0.05; **P < 0.01; ***P < 0.001 by 1-way ANOVA followed by Šídák’s multiple-comparison test.

To determine whether CHI3L1 itself drives profibrotic M2 macrophage differentiation, we evaluated the effect of CHI3L1 on macrophage differentiation using THP-1 monocytes. Recombinant CHI3L1 treatment increased the frequency of CD206^+^CD163^+^ macrophages, and this effect was blocked by either anti-CHI3L1 monoclonal antibody (referred to as FRG) or kasugamycin (KSM), a pan-chitinase inhibitor (Figure 2A). Consistent with our findings in human macrophages, CHI3L1 induced M2 polarization in murine BMDM-derived macrophages, indicating that this response is conserved across species (Supplemental Figure 2). Since studies from our laboratory and others identified PD-L1 (*CD274*) as a critical mediator of CHI3L1-induced immune tolerance and alternative macrophage activation (21, 27), we further assessed PD-L1 expression in CHI3L1-stimulated macrophages. PD-L1 expression was significantly increased in the differentiated macrophages with CHI3L1 stimulation (similar to CD163 and CD206) at both the protein and mRNA levels, and these increases were abrogated by FRG or KSM treatment (Figure 2, B and C). These findings demonstrate that CHI3L1 can directly promote CD206^+^CD163^+^ profibrotic M2 macrophage differentiation with increased expression of PD-L1.

### CHI3L1 enhances TGF-β_1_–stimulated myofibroblast transformation, migration, and invasiveness.

**Figure 3:**
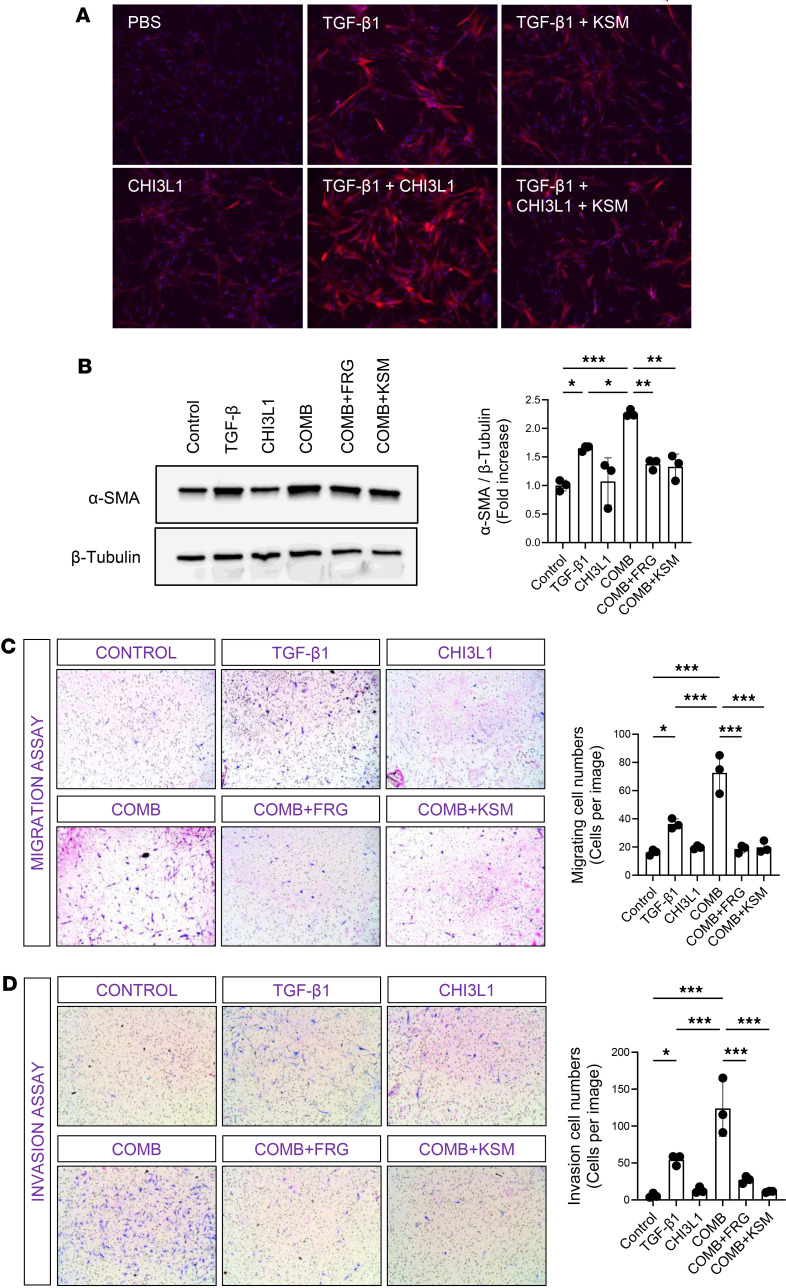
CHI3L1 enhances TGF-β_1_–induced myofibroblast transformation, migration, and invasion. Normal human lung fibroblasts (NHLFs) were treated with recombinant CHI3L1 (500 ng/mL) and/or TGF-β_1_ (5 ng/mL) with or without FRG (250 ng/mL) or KSM (250 ng/mL). (**A** and **B**) α-SMA expression was assessed by immunocytochemistry and Western blotting. (**C** and **D**) Fibroblast migration and invasion assays. In each well, the number of cells that migrated or invaded through the membrane was counted. Original magnification, x200 (**A**); x40 (**C** and **D**). Bar graph in **B** shows densitometric quantification of α-SMA; β-tubulin used as loading control. Data represent mean ± SEM. *P < 0.05; **P < 0.01; ***P < 0.001 by 1-way ANOVA followed by Šídák’s multiple-comparison test. COMB, combined CHI3L1 and TGF-β_1_.

Although fibroblasts and their differentiation into invasive myofibroblasts represent a central driver of PF (1) and CHI3L1 has been implicated in tissue remodeling and fibroproliferative responses (9), whether it directly modulates fibroblast activation in the context of PF has not been well defined. To investigate the role of CHI3L1 in fibroblast-to-myofibroblast transformation, we treated normal human lung fibroblasts (NHLFs) with recombinant CHI3L1, TGF-β_1_, or both. Immunocytochemistry and Western blot analyses revealed minimal increases in α-smooth muscle actin (α-SMA) expression, a marker of myofibroblast transformation, in response to CHI3L1 stimulation alone, while maximal induction was observed with combined CHI3L1 and TGF-β_1_ stimulation (Figure 3, A and B). Fibroblast migration and invasion are hallmark functional properties of activated myofibroblasts and are considered central to progressive tissue scarring in PF. To determine whether CHI3L1 regulates these pathogenic functions, we performed Transwell migration and invasion assays. Consistent with Western blot results, CHI3L1 alone did not affect fibroblast migration or invasion, but it significantly enhanced TGF-β_1_–induced migration and invasion, with maximal effects being observed under combined CHI3L1 and TGF-β_1_ stimulation (Figure 3, C and D). CHI3L1 enhanced the migration and invasion of murine lung–derived fibroblasts, similar to its effects in human lung fibroblasts, indicating that this response is not species specific (Supplemental Figure 3). All of these responses were abrogated by either FRG or KSM treatment, indicating a specific contribution of CHI3L1 to TGF-β_1_–driven myofibroblast transformation, migration, and invasiveness of lung fibroblasts.

### CHI3L1 enhances TGF-β_1_ signaling and its effector functions in lung fibroblasts through the CD44/PD-L1 axis.

**Figure 4:**
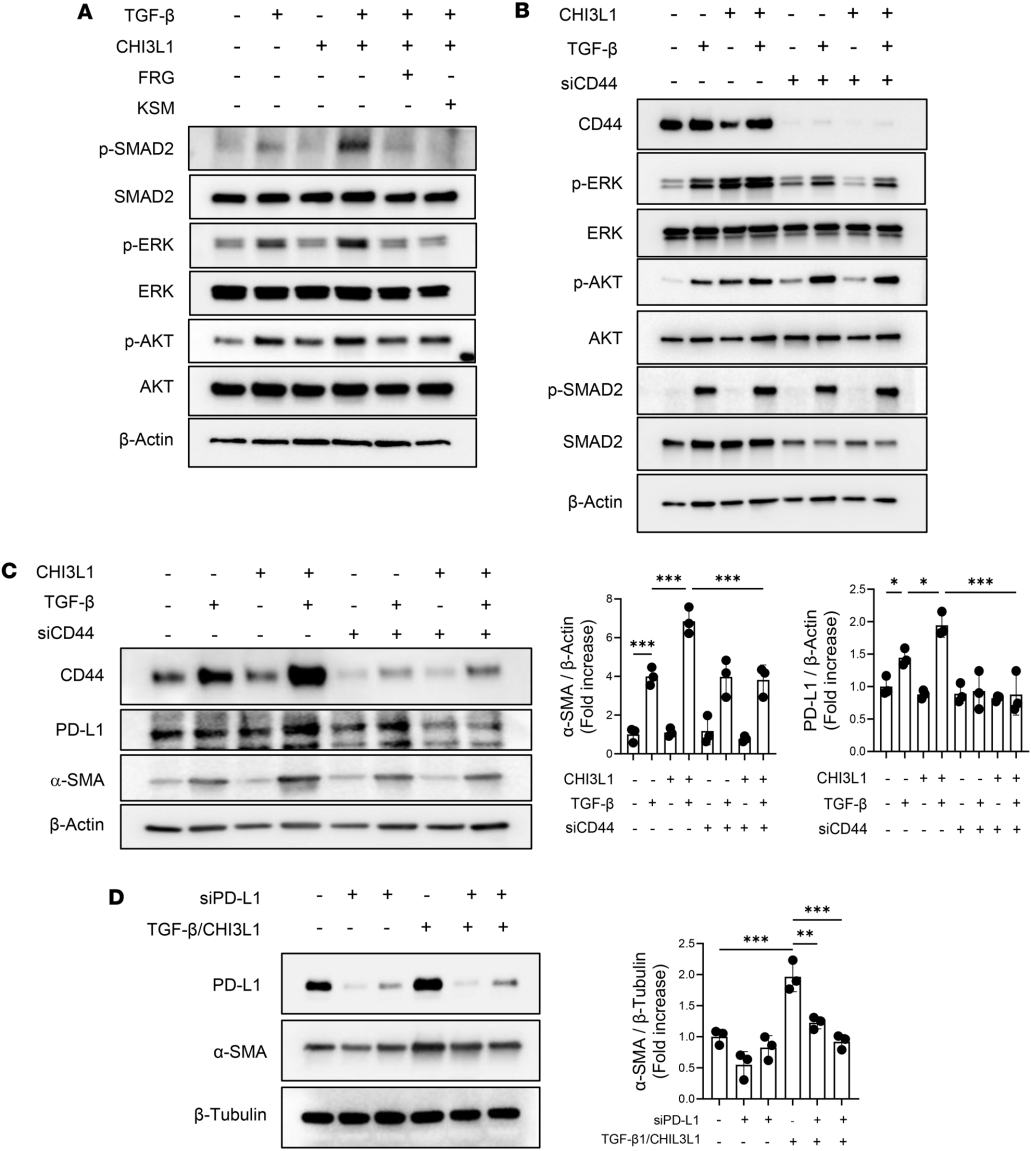
CHI3L1 promotes TGF-β signaling and myofibroblast transformation through a CD44/PD-L1 axis. NHLFs or mouse lung fibroblasts were treated with recombinant CHI3L1 (500 ng/mL) and/or TGF-β_1_ (5 ng/mL) with or without FRG (250 ng/mL) or KSM (250 ng/mL). (**A**) Western blot analysis of SMAD, AKT, and ERK phosphorylation in NHLFs. (**B**) Western blot of the same signaling pathways in mouse lung fibroblasts treated with or without siCD44. (**C**) Western blot of PD-L1 and α-SMA expression in mouse lung fibroblasts stimulated with CHI3L1 and/or TGF-β_1_ in the presence or absence of CD44 silencing. (**D**) Western blot of α-SMA expression in NHLFs with or without siPD-L1. β-Actin was used as a loading control. Panels **A** and **B** show representative results from 3 independent experiments. Bar graphs (**C** and **D**) show densitometric quantification of protein expression (mean ± SEM). *P < 0.05; **P < 0.01; ***P < 0.001 by 1-way ANOVA followed by Šídák’s multiple-comparison test.

To dissect the mechanistic interplay between CHI3L1 and TGF-β_1_ signaling in lung fibroblasts, we first examined the impact of recombinant CHI3L1 on TGF-β_1_–induced signaling in NHLFs or mouse lung fibroblasts. CHI3L1 augmented TGF-β_1_–induced activation of SMAD2 (p-SMAD2), ERK (p-ERK), and AKT (p-AKT), and these effects were abrogated by treatment with FRG antibody or KSM (Figure 4A). Given that CD44 functions as a co-receptor for CHI3L1 and its involvement in TGF-β_1_ signaling (28, 29), we investigated whether the CHI3L1–TGF-β_1_ interaction is mediated through CD44 using CD44-knockdown fibroblasts. Indeed, the effects of CHI3L1 alone and the interaction of CHI3L1 on TGF-β_1_–induced ERK activation were markedly diminished in CD44-silenced mouse lung fibroblasts. In contrast, no notable changes were observed in CHI3L1-induced SMAD2 or AKT activation (Figure 4B). To further define the downstream functional consequences, we evaluated α-SMA and PD-L1 expression, as PD-L1 has been reported to promote fibroblast-to-myofibroblast transformation and tissue remodeling in PF (26, 30). CD44 knockdown abrogated the synergistic effect of CHI3L1 and TGF-β_1_ on α-SMA and PD-L1 induction (Figure 4C). Moreover, PD-L1 knockdown similarly attenuated CHI3L1/TGF-β_1_–induced α-SMA expression, while siRNA control had no effect (Figure 4D). Consistently, CD44 neutralization using an anti-CD44 antibody inhibited the stimulatory effects of CHI3L1 and TGF-β_1_ on PD-L1 and α-SMA expression in NHLFs (Supplemental Figure 4). Collectively, these results suggest that a CHI3L1/CD44/ERK/PD-L1 signaling axis amplifies TGF-β_1_ responses and promotes profibrotic transformation in lung fibroblasts.

### CHI3L1 facilitates profibrotic macrophage-fibroblast crosstalk via the CD44/PD-L1 axis.

**Figure 5:**
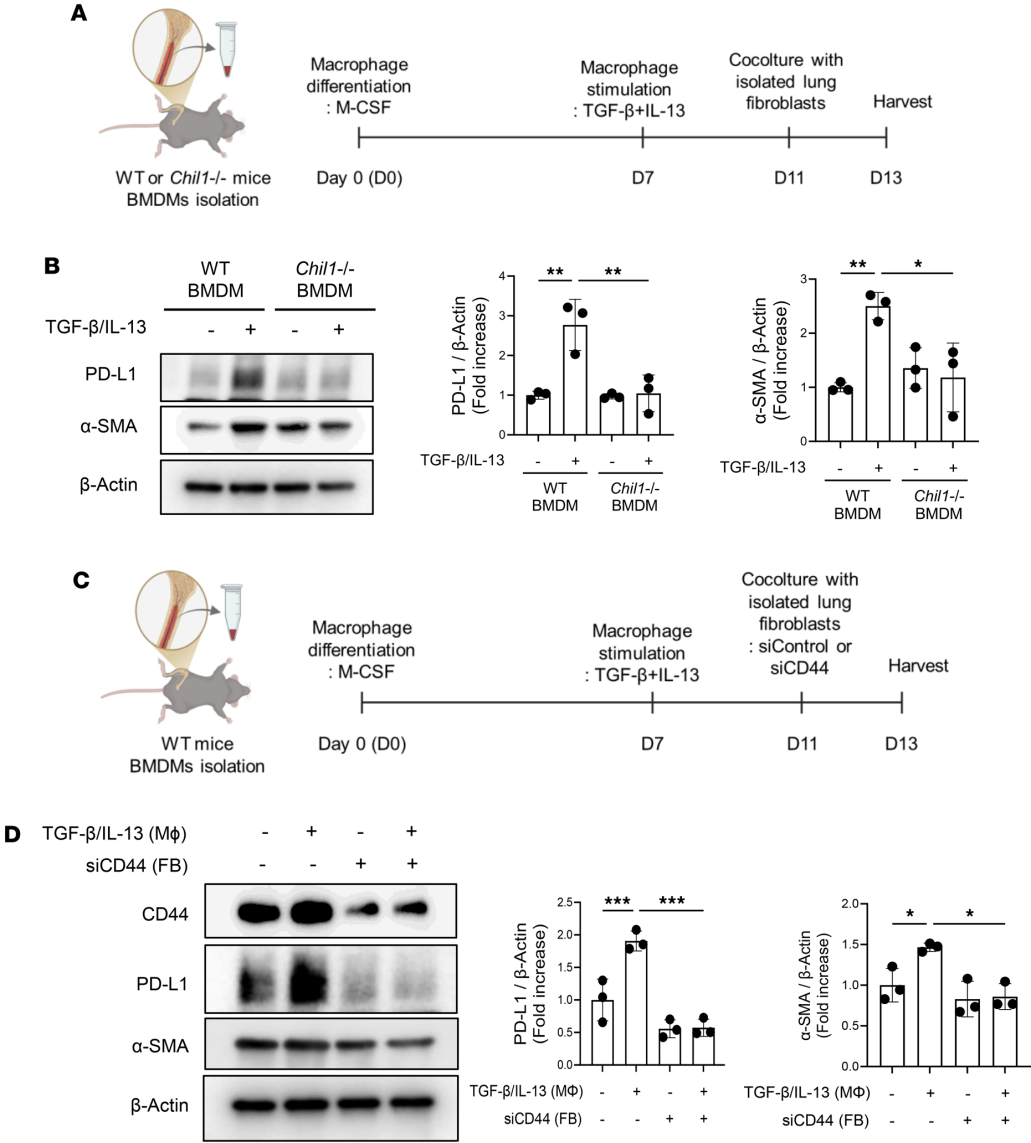
CHI3L1 facilitates profibrotic macrophage-fibroblast crosstalk via a CD44/PD-L1 axis. (**A** and **B**) Coculture of fibroblasts with WT or *Chil1^–/–^* macrophages prestimulated with IL-13 (20 ng/mL) and TGF-β_1_ (5 ng/mL). Western blot of PD-L1 and α-SMA. (**C** and **D**) Coculture of IL-13/TGF-β_1_–stimulated WT macrophages with fibroblasts with and without siCD44. Western blot of PD-L1 and α-SMA. The bar graphs in **B** and **D** represent densitometric quantification of protein expression (mean ± SEM). *P < 0.05; **P < 0.01; ***P < 0.001 by 1-way ANOVA followed by Šídák’s multiple-comparison test. Schematics in **A** and **C** were created with BioRender.com.

Profibrotic macrophage polarization and aberrant fibroblast activation are key contributors to PF (31). To investigate the role of CHI3L1 in macrophage-fibroblast communication, we performed coculture experiments using mouse BMDMs and isolated lung fibroblasts. First, lung fibroblasts were cocultured with BMDMs from WT or CHI3L1-null mice that had been prestimulated with IL-13 and TGF-β_1_ to induce a profibrotic phenotype (Figure 5A). The fibroblast response was assessed by α-SMA and PD-L1 expression. IL-13/TGF-β_1_–stimulated WT macrophages significantly induced both α-SMA and PD-L1 expression in fibroblasts, whereas CHI3L1-null macrophages failed to do so (Figure 5B). In additional experiments, fibroblasts isolated from WT mice, with or without CD44 knockdown, were cocultured with IL-13/TGF-β_1_–stimulated WT macrophages (Figure 5C). CD44-silenced fibroblasts exhibited reduced expression of α-SMA and PD-L1 compared with siControl-transfected fibroblasts (Figure 5D). Collectively, these results establish CHI3L1 as a central mediator of macrophage-fibroblast interactions and demonstrate that the fibroblast CD44/PD-L1 axis is critical for CHI3L1-driven myofibroblast transformation.

### CHI3L1 increases profibrotic M2 macrophages and invasive fibroblasts in bleomycin-challenged lungs.

**Figure 6:**
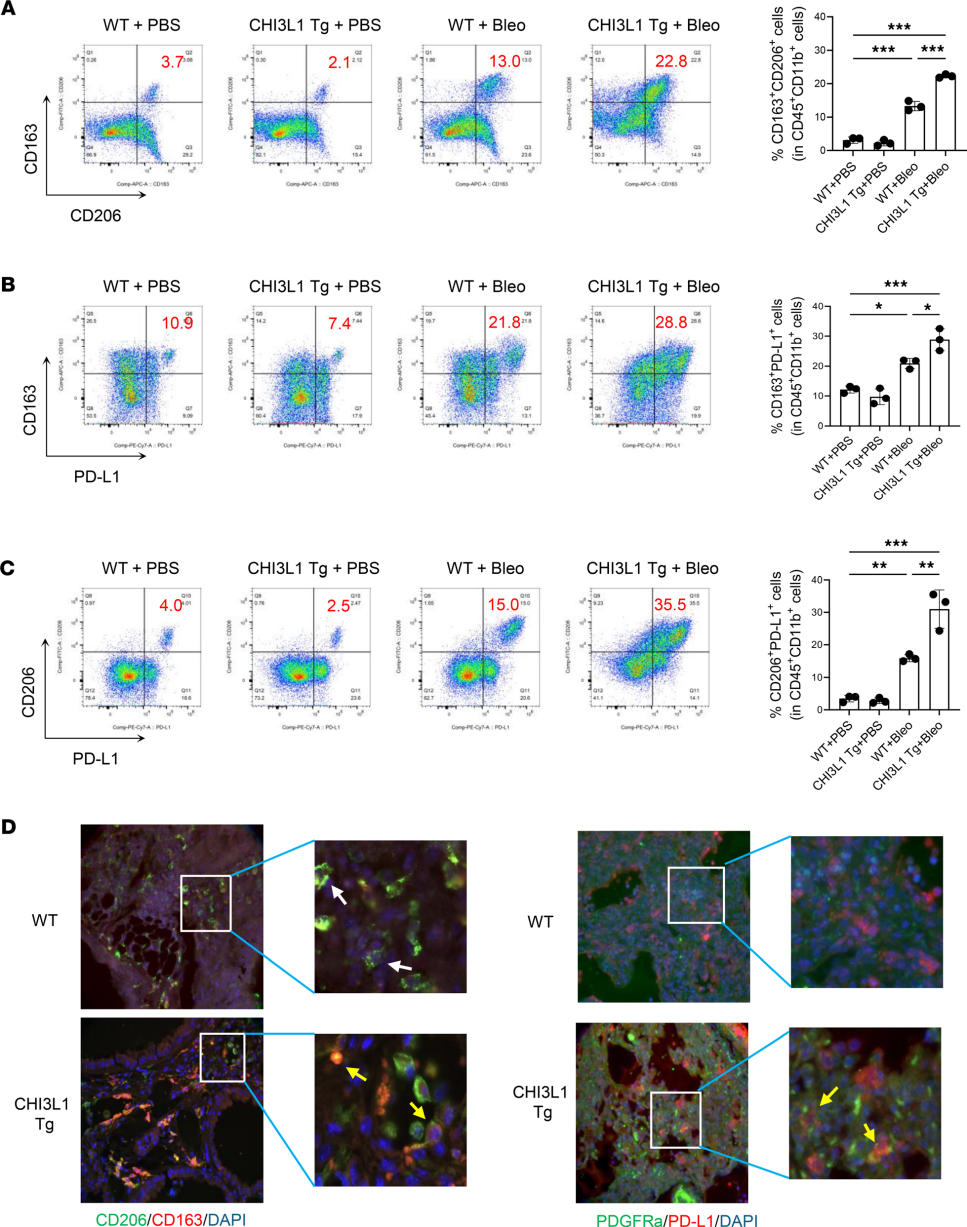
CHI3L1 increases profibrotic macrophages and fibroblasts in bleomycin-challenged lungs. WT and CHI3L1-Tg mice were challenged with PBS or bleomycin. The mice were sacrificed 14 days after bleomycin challenge and analyzed. (**A**–**C**) Flow cytometric analysis of CD206^+^CD163^+^ and PD-L1^+^ macrophages in lungs of WT and CHI3L1-Tg mice. (**D**) IHC of CD206^+^CD163^+^ macrophages and PD-L1^+^PDGFRα^+^ fibroblasts in WT and CHI3L1-Tg lungs. Images were acquired at a total magnification of ×200; Insets represent ×3 digitally enlarged regions of the corresponding images. Flow cytometry and IHC data in **A**–**C** are representative of at least 3 independent experiments. *P < 0.05; **P < 0.01; ***P < 0.001 by 1-way ANOVA followed by Šídák’s multiple-comparison test.

To evaluate the in vivo role of CHI3L1 in driving profibrotic macrophage responses, WT and CHI3L1-overexpressing transgenic (CHI3L1-Tg) mice were treated with bleomycin. In WT lungs, bleomycin exposure led to increased accumulation of CD206^+^CD163^+^ and SiglecF^+^CX3CR1^+^ profibrotic macrophages, and these responses were further augmented in CHI3L1-Tg mice (Figure 6A and Supplemental Figure 5). Notably, the number of PD-L1^+^CD206^+^ and PD-L1^+^CD163^+^ macrophages was significantly elevated in CHI3L1-Tg lungs compared with WT (Figure 6, B and C). Immunohistochemical analysis further confirmed the expansion of CD206^+^CD163^+^ macrophages and revealed a notable increase in PD-L1^+^PDGFRα^+^ mesenchymal cells, including fibroblasts, in the lungs of CHI3L1-Tg mice compared with controls (Figure 6D). These findings indicate that CHI3L1 enhances the development of PD-L1–expressing profibrotic macrophages and invasive fibroblasts in the setting of bleomycin-induced lung injury. In addition, we observed a marked increase in total PD-1^+^ cells and CD45^+^PD-1^+^ immune cells, including CD45^+^CD11c^+^PD-1^+^ macrophages in the lungs of CHI3L1-Tg mice compared with WT (Supplemental Figure 6), suggesting that enhanced PD-1–PD-L1 interactions contribute to the immunosuppressive and fibrotic microenvironment orchestrated by CHI3L1. These data suggest that CHI3L1-driven immune-fibrotic circuits are reproduced in vivo, reinforcing its central pathogenic role.

### A bispecific anti-CHI3L1×PD-1 antibody consolidates the efficacy of combination therapy in bleomycin-induced lung fibrosis.

**Figure 7:**
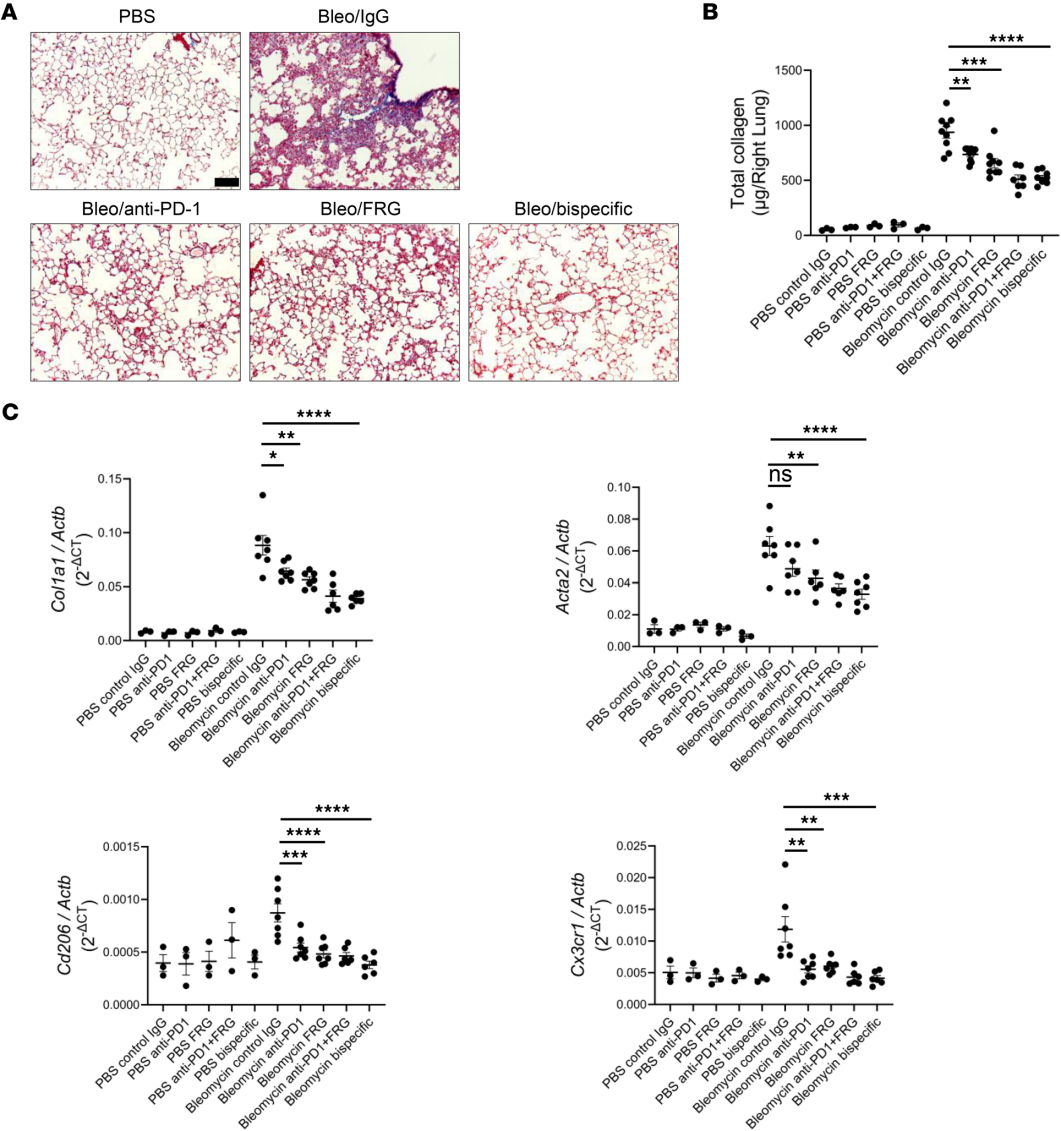
Anti-CHI3L1, anti-PD-1, and bispecific antibodies attenuate bleomycin-induced lung fibrosis. WT mice were challenged intratracheally with PBS or bleomycin and subsequently treated with FRG, anti-PD-1 antibody, or a bispecific anti-CHI3L1×PD-1 antibody, either individually or in combination. (**A**) Histological assessment and Masson’s trichrome staining of lung tissues from bleomycin-challenged mice treated with FRG, anti-PD-1, or the bispecific antibody. Scale bar: 100 μm. (**B**) Quantification of total lung collagen content. (**C**) qRT-PCR of the expression of fibrotic markers (*Col1a1*, *Acta2*) and profibrotic macrophage markers (*Cd206*, *Cx3cr1*). The values in **B** and **C** are mean ± SEM. *P < 0.05; **P < 0.01; ***P < 0.001; ****P < 0.0001 by 1-way ANOVA followed by Šídák’s multiple-comparison test.

Because our studies demonstrated that CHI3L1 plays a key role in profibrotic macrophage activation and fibroblast responses through the CD44/PD-1/PD-L1 axis, we next evaluated the in vivo therapeutic potential of mono- or bispecific antibodies targeting CHI3L1 and the PD-1/PD-L1 axis in a bleomycin-induced PF mouse model. Mice treated with bleomycin and isotype control (IgG) showed significant increases in pulmonary collagen content and fibrosis-related gene expression of *Col1a1*, *Acta2* (encoding α-SMA), *Cd206*, and *Cx3cr1* (Figure 7). Treatment with anti-CHI3L1 or anti-PD-1 monotherapies partially reduced these fibrotic responses, but the effects were limited. In contrast, both the bispecific anti-CHI3L1×PD-1 antibody and the combination of anti-PD-1 plus anti-CHI3L1 (FRG) antibodies produced the most pronounced attenuation of fibrosis, as evidenced by histological analysis, collagen quantification, and transcriptional suppression of fibrotic (*Col1a1* and *Acta2*) and profibrotic macrophage (*Cd206* and *Cx3cr1*) markers (Figure 7, A–C). FACS analysis further corroborated these findings, showing reduced frequencies of profibrotic macrophages in mice treated with the bispecific or combination therapies (Supplemental Figure 7). Notably, the bispecific antibody achieved efficacy comparable to combination treatment while clearly outperforming monotherapies. Interestingly, we also noted that anti-CHI3L1 (FRG) or anti-PD-1 antibodies significantly altered the number of CD45^+^ cells infiltrated in the lung, whereas treatment with the bispecific antibody produced the greatest reduction (Supplemental Figure 8). These findings suggest that these antibodies exert antiinflammatory effects, in addition to modulating the functional phenotypes of macrophages and fibroblasts, as described in this study. Together, these results demonstrate that dual blockade of CHI3L1 and PD-1 can effectively suppress fibrotic remodeling in vivo, and they establish the bispecific antibody as a therapeutically attractive strategy that consolidates the benefits of combination therapy into a single molecular format.

### Increased CHI3L1 and PD-1 expression and spatial interaction between CHI3L1^+^ macrophages and CD44^+^ fibroblasts in the lungs of patients with IPF compared with controls.

**Figure 8:**
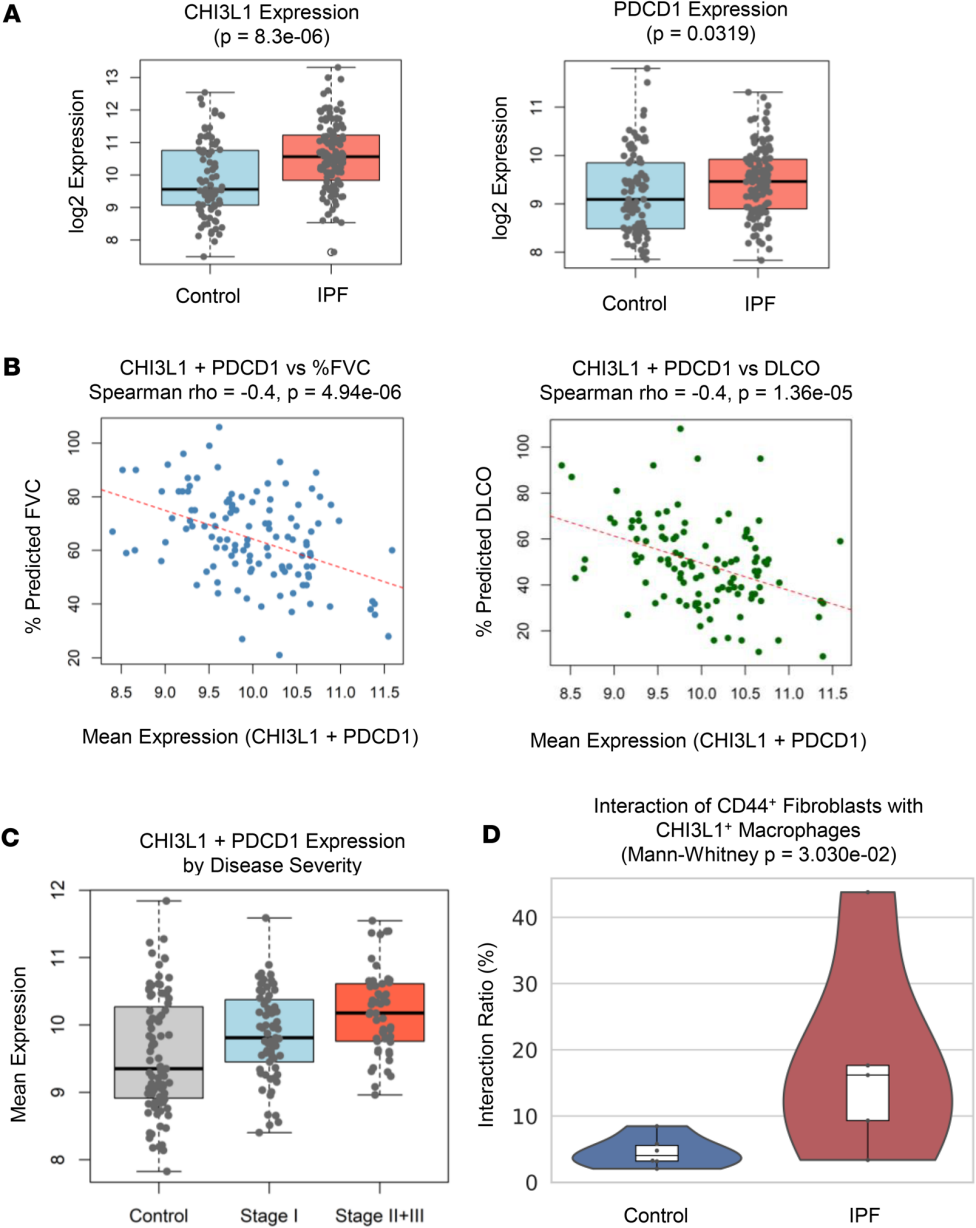
Increased CHI3L1 and PD-1 expression and spatial interaction between CHI3L1^+^ macrophages and CD44^+^ fibroblasts in IPF lungs. (**A**) Reanalysis of the Lung Tissue Research Consortium microarray dataset (NCBI GEO GSE47460) showing significantly elevated *CHI3L1* and *PDCD1* (PD-1) expression in IPF lungs. (**B**) Coexpression of *CHI3L1* and *PDCD1* inversely correlated with pulmonary function indices, percentage predicted FVC, and DLCO. (**C**) Mean expression of *CHI3L1* and *PDCD1* progressively increased from healthy controls to IPF patients and was further stratified by disease severity. Pairwise comparisons showed significant differences between control versus stage I, stage I versus stage II+III, and control versus stage II+III. (**D**) Spatial transcriptomic dataset (6 controls and 5 IPF) revealed higher interaction ratios between CHI3L1^+^ macrophages and CD44^+^ fibroblasts within 20 μm in IPF lungs (*P* < 0.05 by Mann-Whitney *U* test). For the box-and-whisker plots in panels **A** and **C**, boxes represent the interquartile range (25th–75th percentile), center lines indicate the median, and whiskers extend to the most extreme values within 1.5 × the interquartile range. Individual data points are overlaid where shown. In panel **D**, violin plots depict the distribution of the data, with embedded box plots showing the interquartile range and median; individual data points are shown by the overlaid swarm plot.

To assess the human relevance of our findings, we analyzed publicly available RNA profiling datasets from patients with interstitial lung disease (ILD) and controls. First, we examined a large microarray dataset (NCBI GEO GSE47460) (32) from the Lung Tissue Research Consortium (LTRC), which includes samples from 254 ILD patients and 108 controls. According to the dataset metadata, patients were categorized based on clinical history, CT imaging, and surgical pathology. In this dataset, *CHI3L1* and *PDCD1* (PD-1) transcript levels were significantly elevated in the lungs of IPF patients compared with controls (Figure 8A). Notably, coexpression of *CHI3L1* and *PDCD1* was inversely correlated with pulmonary function indices, including percentage predicted forced vital capacity (FVC) and diffusion capacity of the lungs for carbon monoxide (DLCO) (Figure 8B), suggesting functional relevance of these genes in disease outcomes. Moreover, mean *CHI3L1* and *PDCD1* expression progressively increased from healthy controls to IPF patients, with further stratification by disease severity (Figure 8C). Pairwise analyses showed significant differences between control versus stage I (*P* = 0.016), stage I versus stage II+III (*P* = 0.017), and control versus stage II+III (*P* = 6.6 × 10^–6^), indicating that coexpression of these immune-fibrotic genes reflects both disease presence and progression. To further examine spatial relationships, we analyzed a recently published spatial transcriptomic dataset that included samples from 6 healthy individuals and 5 IPF patients (33). Spatial interaction analysis demonstrated that CHI3L1^+^ macrophages and CD44^+^ fibroblasts had significantly higher interaction ratios in IPF lungs compared with controls (Figure 8D). These findings suggest that close interactions between this macrophage subset and fibroblasts may contribute to the pathogenic fibro-inflammatory circuits that drive PF.

## Discussion

The present study shows that CHI3L1 is a critical mediator of profibrotic macrophage activation and invasive myofibroblast transformation, primarily through CD44 receptor–mediated upregulation of PD-L1 expression. Our findings demonstrate that CHI3L1 coordinates both immune and stromal responses by promoting M2 macrophage differentiation and amplifying TGF-β_1_–driven invasive properties of myofibroblasts. These results highlight CHI3L1-driven crosstalk that promotes the progression of PF and suggest potential therapeutic targets for intervention.

We further demonstrate that CHI3L1 is both necessary and sufficient to drive alternative (M2) macrophage activation in response to profibrotic cytokines such as IL-13 and TGF-β_1_. Genetic deletion of CHI3L1 markedly reduced the expression of canonical M2 markers (CD206 and CD163) and profibrotic markers (CX3CR1 and SiglecF) in BMDMs and in vivo bleomycin models, underscoring its essential role in skewing the macrophage phenotype toward a profibrotic state. Moreover, in human THP-1 cells, recombinant CHI3L1 induced M2 differentiation and PD-L1 expression, a key immune checkpoint molecule that suppresses T cell responses and promotes fibrosis. These CHI3L1-driven effects were abrogated by either FRG or KSM, a pan-chitinase inhibitor, suggesting both therapeutic and mechanistic relevance.

CHI3L1 is elevated in the serum and tissues from IPF patients, and its role in the pathogenesis of PF has been demonstrated in animal models (9). However, the precise mechanism by which CHI3L1 regulates PF has remained unclear. It is noteworthy that CHI3L1 is involved in IL-13/TGF-β_1_–induced M2 and profibrotic macrophage differentiation, suggesting its essential role in PF. Consistent with this, recent single-cell RNA-seq analysis on the lungs of IPF patients demonstrated that CHI3L1 is highly expressed in profibrotic macrophages, called IPF-expanded macrophage (IPFeMϕ), predominantly observed in IPF patients (34). Additional analysis of publicly available single-cell RNA-seq data from other IPF or ILD cohorts confirmed that CHI3L1-expressing macrophages are enriched in lungs of IPF patients but not in normal, COPD, or other lung diseases (35–37). Together, these findings support CHI3L1 as a driver of profibrotic macrophage activation in human IPF.

Our findings reveal that CHI3L1 drives PD-L1 induction during M2 macrophage polarization, establishing a mechanistic link between immune checkpoint signaling and fibrosis. CHI3L1 promoted the differentiation of CD206^+^CD163^+^ macrophages with concomitant PD-L1 upregulation, and this effect was abrogated by FRG or KSM. This aligns with prior studies showing that PD-L1 not only mediates immune suppression but also reinforces M2-like macrophage phenotypes (38, 39) and contributes to fibroblast-to-myofibroblast transformation and ECM remodeling (24, 30, 40). Collectively, these data position the CHI3L1/PD-L1 axis as a central node linking immune suppression with fibrotic remodeling and underscore its potential as a therapeutic vulnerability in PF.

Beyond macrophages, we found that CHI3L1 potentiates TGF-β–induced myofibroblast transformation, migration, and invasiveness in lung fibroblasts. Mechanistically, CHI3L1 amplified TGF-β–mediated phosphorylation of ERK, AKT, and SMAD2, with ERK activation being particularly dependent on CD44, a known co-receptor of CHI3L1 (28). Our study demonstrates that CD44 is essential for this CHI3L1/TGF-β–induced myofibroblast transformation. CD44 knockdown or neutralization inhibited CHI3L1/TGF-β synergy in promoting α-SMA and PD-L1 expression, as did PD-L1 knockdown. These data define what we believe is a novel CHI3L1/CD44/ERK/PD-L1 signaling axis that integrates immunoregulatory and fibrogenic pathways within fibroblasts. This observation is consistent with previous reports implicating CD44 in PF (41–45) and suggests that the CD44/PD-L1 axis may represent a therapeutic target for blocking CHI3L1/TGF-β–driven fibroblast activation.

In the development and progression of PF, the importance of intercellular crosstalk between immune and non-immune cells, including macrophages, epithelial cells, and fibroblasts, has been well established (31, 46, 47). Here, we show that CHI3L1 mediates direct crosstalk between profibrotic macrophages and fibroblasts through coculture experiments. Macrophages preactivated with IL-13 and TGF-β_1_ induced robust profibrotic effects in fibroblasts in a CHI3L1- and CD44-dependent manner. This bidirectional loop likely amplifies and sustains the fibrotic cascade, with PD-L1 serving as a shared effector molecule in both cell types. In vivo, lungs of CHI3L1-Tg mice exhibited a marked increase in PD-L1^+^ M2 macrophages and PD-L1^+^PDGFRα^+^ fibroblasts following bleomycin challenge, accompanied by an expansion of CD45^+^PD-1^+^ immune populations. These findings suggest that CHI3L1 contributes to immune checkpoint engagement and immune evasion within fibrotic lungs, a phenomenon previously described in cancer biology (21, 27, 48) but less well characterized in fibrotic disease. Together, our results identify a CHI3L1/CD44/PD-L1 axis as a key mediator of macrophage-fibroblast crosstalk and define a pathogenic feedback loop that sustains fibrosis. This axis offers potential intervention points to disrupt progressive tissue remodeling.

It is also important to note that epithelial cells, particularly alveolar type II (AT2) cells, represent a major source of CHI3L1, and dysregulation of epithelial CHI3L1 may substantially contribute to profibrotic lung injury and repair responses (9). Future studies examining macrophage-epithelial and macrophage-epithelial-fibroblast interactions in the context of dysregulated CHI3L1 signaling and AT2 cell integrity will provide important mechanistic insights into the role of CHI3L1 in progressive PF.

Notably, therapeutic blockade of CHI3L1 and PD-1 using bispecific antibodies or combined monotherapies markedly attenuated fibrosis severity, reduced profibrotic macrophage activation, and diminished the expression of fibrotic markers. While anti-CHI3L1 or anti-PD-1 monotherapies produced only partial benefit, both the bispecific anti-CHI3L1×PD-1 antibody and the combination of individual antibodies achieved the most pronounced effects. The bispecific antibody, therefore, matched the efficacy of combination therapy while clearly outperforming single-agent treatments. Beyond efficacy, the bispecific format offers potential translational advantages, including simplified dosing, the potential for improved pharmacokinetics, and reduced manufacturing complexity. These features position the bispecific antibody as a particularly attractive therapeutic approach that consolidates the benefits of dual blockade into a single molecular entity, underscoring its promise for clinical development in PF.

In addition to our mechanistic findings in vitro and in vivo, our analysis of large publicly available human transcriptomic datasets provides important clinical context. We observed that *CHI3L1* and *PDCD1* (PD-1) transcript levels were significantly elevated in IPF patients compared with controls, and that their coexpression inversely correlated with lung function indices, including FVC and DLCO (32). Notably, expression progressively increased with disease severity, suggesting that the CHI3L1/PD-1 axis is not only a marker of disease presence but also a driver of disease progression. These observations are consistent with prior reports linking CHI3L1 to IPF severity and extend them by demonstrating its close association with PD-1, a key immune checkpoint molecule that promotes tolerance and fibrotic remodeling (8, 9, 15). Moreover, spatial transcriptomic analyses demonstrated increased proximity of CHI3L1^+^ macrophages and CD44^+^ fibroblasts in IPF lungs compared with controls (33). This finding suggests that CHI3L1 contributes to pathogenic macrophage-fibroblast crosstalk within the fibrotic niche. Together with our coculture and in vivo findings, these human data highlight CHI3L1 as a central mediator of immune-fibrotic circuits in IPF and reinforce the rationale for simultaneously targeting CHI3L1 and PD-1/PD-L1.

While our study supports the therapeutic value of dual CHI3L1 and PD-1/PD-L1 blockade, several limitations warrant further investigation. First, although our in vitro and in vivo models elucidate the role of CHI3L1 in macrophage-fibroblast crosstalk, the precise molecular mechanisms downstream of PD-L1 that contribute to myofibroblast transformation remain incompletely understood. In particular, dissecting whether PD-L1 functions in its membrane-bound versus soluble form, and how these isoforms interact with PD-1 expressed on macrophages or T cells, will be essential for understanding the full scope of PD-1/PD-L1–mediated immune modulation in the fibrotic lung. Second, while bispecific antibody therapy targeting CHI3L1 and PD-1 showed enhanced antifibrotic efficacy in our preclinical model, comprehensive evaluation of long-term safety, pharmacokinetics, and immunogenicity is required prior to translation into clinical settings. Future studies should include progressive and persistent fibrosis models and expanded immune profiling to better characterize the durability and specificity of the therapeutic response. Ultimately, integrating mechanistic insights with rigorous preclinical testing will be critical for advancing CHI3L1-based immunotherapies for PF and related fibrotic diseases.

In current studies, we observed increased numbers of CHI3L1^+^PD-L1^+^ macrophages and PD-L1^+^ fibroblasts at the late stage (day 14) following bleomycin challenge, corresponding to the fibroproliferative phase of the model. These findings suggest that the emergence and expansion of these profibrotic cell populations occur during the fibroproliferative repair phase. A more detailed temporal analysis of these populations will therefore be important to better define the dynamics of this process and to determine the optimal window for therapeutic intervention in future studies.

Taken together, our study defines CHI3L1 as a key pathogenic mediator that bridges immune activation, stromal remodeling, and immune checkpoint signaling in PF. These findings expand our understanding of CHI3L1 beyond its known roles in cancer and infection, and offer new therapeutic avenues for fibrotic lung diseases, including IPF, where current treatment options remain limited. Future studies should explore CHI3L1’s interactions with other immune and stromal cell populations, assess its utility as a biomarker of disease progression, and validate the efficacy of CHI3L1-targeted therapies in diverse models of organ fibrosis.

## Methods

Detailed information on all antibodies, reagents, recombinant proteins, and chemicals used in this study is provided in Supplemental Table 1.

### Sex as a biological variable.

Both male and female mice were used in this study, and animals were age- and sex-matched between experimental groups. Data from both sexes were combined for analysis.

### Mice and animal models.

WT, CHI3L1-null (*Chil1*^–/–^), and CHI3L1-Tg mice were generated and characterized in our laboratory as previously described (20, 49). Experiments were conducted using age- (8–10 weeks old) and sex-matched WT and CHI3L1-Tg mice.

### Cell lines.

Human monocytic THP-1 cells (ATCC, TIB-202) were cultured in RPMI-1640 supplemented with 10% heat-inactivated FBS and antibiotics (1% penicillin-streptomycin).

### Primary cells.

NHLFs (ATCC) were cultured in FBM Fibroblast Growth Basal Medium with FGM-2 Fibroblast Growth Medium-2 SingleQuots Supplements and Growth Factors. Murine primary lung fibroblasts were isolated and cultured as described previously (50) and cultured in DMEM containing 10% FBS and antibiotics.

### BMDMs.

BMDMs were isolated from the femurs of WT and *Chil1*^–/–^ mice as previously described (51). Bone marrow was cultured in complete DMEM medium with 10% FBS and 25 ng/mL M-CSF for 7 days to differentiate into macrophages. Macrophages (1 × 10^6^/well in 6-well plates) were stimulated with recombinant IL-13 (20 ng/mL) and TGF-β_1_ (5 ng/mL), or IFN-γ (20 ng/mL) for 72 hours and subjected to further evaluation.

### THP-1 cell differentiation and macrophage activation.

Human monocytic THP-1 cells were differentiated into macrophages with phorbol 12-myristate 13-acetate (PMA; 100 ng/mL) for 24 hours and rested with complete medium for 24 hours. The differentiated cells were then treated with recombinant CHI3L1 (500 ng/mL) with or without anti-CHI3L1 antibody (FRG; 250 ng/mL) or kasugamycin (KSM; 250 ng/mL). After 72 hours, flow cytometry was performed to assess the expression of M2 markers (CD163^+^, CD206^+^), and the PD-L1 levels were analyzed by qPCR and Western blot.

### Flow cytometry.

Single-cell suspensions from whole mouse lungs were prepared using the Lung Dissociation Kit (Miltenyi Biotec) according to the manufacturer’s instructions, followed by RBC lysis. Cells were stained with fluorescently conjugated antibodies (see Supplemental Table 1). Data were acquired using the BD FACSAria III and the Cytek Aurora and analyzed with FlowJo software (v10). Gating strategy of the macrophages and other cells employed in this study is illustrated in Supplemental Figure 9.

### Double-label fluorescent immunohistochemistry.

Formalin-fixed, paraffin-embedded (FFPE) lung tissue blocks were sectioned into 5-μm-thick slices and mounted onto glass slides. Sections were deparaffinized, rehydrated, and subjected to heat-induced epitope retrieval using a steamer and antigen unmasking solution (Abcam, citrate buffer, pH 6.0) for 30 minutes. Blocking was performed with serum-free protein blocking solution (Agilent/Dako) for 10 minutes at room temperature. Slides were incubated overnight at 4°C with fluorophore-conjugated primary antibodies (see Supplemental Table 1). After washing, fluorescently labeled secondary antibodies were applied for 1 hour at room temperature. Sections were mounted with VECTASHIELD with DAPI (Vector Laboratories).

### RNA extraction and semiquantitative real-time qPCR.

Total RNA was isolated using QIAzol reagent (Qiagen) followed by RNA purification with the RNeasy Mini Kit (Qiagen) according to the manufacturer’s instructions. Reverse transcription and real-time PCR were performed as previously described (20). Ct values for target genes were normalized to internal housekeeping genes *GAPDH* and *Actb*. Primer sequences used in the real-time qPCR are the following: *CD274*-S: CAAAGAATTTTGGTTGTGGA, *CD274*-AS: AGCTTCTCCTCTCTCTTGGA; *GAPDH*-S: GAAGGTCGGAGTCAACGGATT, *GAPDH*-AS: CGCTCCTGGAAGATGGTGAT; *Col1a1*-S: GCTCCTCTTAGGGGCCACT, *Col1a1*-AS: CCACGTCTCACCATTGGGG, *Acta2*-S: TCAGCGCCTCCAGTTCCT, *Acta2*-AS: AAAAAAAACCACGAGTAACAAATCAA, *Cd206*-S: CTCTGTTCAGCTATTGGACGC, *Cd206*-AS: TGGCACTCCCAAACATAATTTGA; *CX3CR1*-S: TCTTCACGTTCGGTCTGGTG, *CX3CR1*-AS: AGGATGAGTCTGACGGCTCT; *Actb*-S: GGCTGTATTCCCCTCCATCG, *Actb*-AS: CCAGTTGGTAACAATGCCATGT.

### Western blotting.

Western blots were used according to standard protocols as previously described (21). Antibodies against p-SMAD2, p-ERK, p-AKT, α-SMA, SMAD2, ERK, AKT, CD44, CD163, CD206, PD-L1, β-actin, β-tubulin, and mouse PD-L1 were used (see Supplemental Table 1). Signals were detected by SuperSignal West Pico and Femto substrates and quantified by ImageJ.

### Fibroblast migration assays.

NHLFs were pretreated with recombinant TGF-β_1_ (5 ng/mL), recombinant CHI3L1 (500 ng/mL), FRG, or KSM for 48 hours. Following stimulation, 3 × 10^4^ cells were seeded into the upper chamber of 24-well Transwell insert with 8-μm-pore membranes (Corning, 353097) in serum-free DMEM. The lower chamber was filled with DMEM containing 10% FBS as a chemoattractant. After 24 hours, non-migrating cells on the upper surface of membrane were removed using a cotton swab. Migrated cells on the lower surface were stained with Kwik-Diff solution (Thermo Fisher Scientific) and quantified.

### Fibroblast invasion assays.

NHLFs were pretreated as in the migration assay. Subsequently, 8 × 10^4^ cells were seeded into 8-μm-pore BioCoat Matrigel invasion chambers (Corning, 354480) in serum-free DMEM. The lower chamber was filled with serum-free DMEM containing recombinant platelet-derived growth factor-BB (rPDGF-BB; 10 ng/mL) as a chemoattractant. After 24 hours, non-invading cells on the upper surface of membrane were removed. Invading cells on the lower surface were stained with Kwik-Diff solution and quantified.

### siRNA transfection in fibroblasts.

NHLFs or primary lung fibroblasts were transfected with siRNA using Lipofectamine RNAiMAX (Thermo Fisher Scientific). siRNA targeting human PD-L1 (100 nM; Bioneer, 29126-1 and 29126-2) and mouse CD44 (100 nM; Bioneer, 12505-1 and 12505-2) or control siRNA (100 nM; Bioneer, SN-1003) were used. The efficiency of PD-L1 or CD44 silencing was evaluated by Western blotting.

### Coculture studies.

Coculture experiments were performed to assess the interaction between macrophages and fibroblasts. WT or *Chil1*^–/–^ BMDMs were stimulated with recombinant IL-13 (20 ng/mL) and recombinant TGF-β_1_ (5 ng/mL) for 72 hours. Primary lung fibroblasts were then cocultured with macrophages, and after 48 hours, the expression of α-SMA and PD-L1 in the fibroblasts was analyzed by Western blotting. To evaluate the effect of CD44 on fibroblasts from stimulated macrophages, fibroblasts transfected with siCD44 or control siRNA were coincubated with IL-13/TGF-β_1_–stimulated WT macrophages for 48 hours.

### Bleomycin-induced PF and histological analysis.

WT and CHI3L1-Tg mice were given doxycycline in drinking water for 7 days, followed by intratracheal injection of bleomycin (2 U/kg body weight). Fourteen days after bleomycin administration, lung tissues were collected for FACS or histological analysis. Hematoxylin and eosin (H&E) staining was performed to assess lung architecture, and Masson’s trichrome staining was used to assess collagen deposition. A Sircol collagen assay was used to measure total collagen accumulation in the lung, as previously described (20).

### Therapeutic treatment with antibodies.

Mice intratracheally challenged with bleomycin were treated intraperitoneally with anti-CHI3L1 antibody (FRG; 8 mg/kg), anti-PD-1 (8 mg/kg), a combination of both antibodies, or a bispecific anti-CHI3L1×PD-1 antibody (8 mg/kg). Antibody treatments were administered every other day from day 7 to day 14 following bleomycin challenge. We reasoned that initiating CHI3L1 neutralization and other antibody treatment at day 7 would be optimal to interrupt the profibrotic repair responses mediated by CHI3L1 signaling based on the previous studies (9). Fibrosis was evaluated by histology and gene expression analysis (*Col1a1* and *Acta2*, *Cd206*, and *Cx3cr1*). FACS analysis was used to assess the activation of profibrotic macrophages.

### Correlation analysis between gene expression and lung function parameters.

Using IPF cases from GSE47460 with available percentage predicted FVC and percentage predicted DLCO, we computed a 2-gene score as the mean of log_2_-transformed *CHI3L1* (A_23_P137665) and *PDCD1* (A_23_P136405) intensities. Associations with FVC and DLCO were assessed by Spearman’s rank correlation (R cor.test, 2-sided; *P* < 0.05). For visualization, scatter plots were generated with a linear regression line and a LOESS smoother. Single-gene analyses (*CHI3L1*, *PDCD1*) yielded directionally consistent results.

### Spatial interaction analysis.

Spatial transcriptomics data from Mayr et al. (33) were analyzed to evaluate potential physical interactions between CHI3L1^+^ macrophages and CD44^+^ fibroblasts. Cells were annotated by type, and CHI3L1^+^ or CD44^+^ subsets were defined by expression thresholds. For each sample, the spatial coordinates of CHI3L1^+^ macrophages and CD44^+^ fibroblasts were extracted. Using a k-d tree algorithm, we computed the nearest-neighbor distances from each CHI3L1^+^ macrophage to the closest CD44^+^ fibroblast. Cells were considered “interacting” if this distance was 20 μm or less, consistent with previous spatial transcriptomics studies (52, 53). The interaction ratio was defined as the proportion of CD44^+^ fibroblasts that were spatially proximal (≤20 μm) to any CHI3L1^+^ macrophage. Interaction ratios were compared between IPF and healthy samples using the Mann-Whitney *U* test. Additionally, all pairwise distances were aggregated and subjected to the Mann-Whitney *U* test for group-level comparisons. Such nearest-neighbor and distance-threshold-based proximity analyses are widely used in spatial transcriptomics studies (54, 55). All spatial analyses were performed in Python.

### Statistics.

All statistical analyses were conducted using GraphPad Prism software. Comparisons between groups were made using 2-tailed Student’s *t* tests. For multiple group comparisons, 1-way ANOVA followed by appropriate post hoc tests was performed. The Mann-Whitney *U* test was used to test the distributions of 2 independent samples when the data were not normally distributed or were at least ordinal. Data are presented as mean ± SEM. Statistical details of each experiment are provided in the figure legends. Significance was set at a *P* value of less than 0.05.

### Study approval.

All animal experiments were reviewed and approved by the Institutional Animal Care and Use Committee (IACUC) at Brown University (Providence, Rhode Island, USA; protocol 22-11-0004).

### Data availability.

All data supporting the findings of this study are available within the paper and its supplemental material. Values for all data points in graphs are reported in the Supporting Data Values file. Public transcriptomic datasets used in this study are available from the NCBI Gene Expression Omnibus (GEO) under accession number GSE47460 and from Zenodo (https://zenodo.org/records/10012934). Spatial interaction analyses were performed using standard Python libraries (Scanpy, SciPy, and Seaborn) with commonly used nearest-neighbor algorithms; no custom code was generated. Additional raw data are available from the corresponding author upon reasonable request.

## Author contributions

CGL conceptualized and designed the study. HSJ, TS, JHL, SK, BM, and YZ performed experiments and analyzed data. HSJ and TS contributed to data curation and statistical analysis. CGL, HSJ, and TS wrote the original draft of the manuscript. CGL supervised the overall experimental process. JAE and SJS provided critical comments on the experimental data. All authors reviewed and edited the manuscript and approved the final version for submission.

## Conflict of interest

JAE is a cofounder of Elkurt Therapeutics and Sakonnet Biomedical, which develop inhibitors of 18 glycosyl hydrolases as therapeutics. JAE, CGL, and SK have composition of matter and use patents relating to antibodies against CHI3L1 (US Patent 10,253,111, “Methods and Compositions Relating to Anti-CHI3L1 Antibody Reagents”). CGL serves as a consultant for siRNAgen Inc., which develops RNA therapeutics.

## Funding support

This work is the result of NIH funding, in whole or in part, and is subject to the NIH Public Access Policy. Through acceptance of this federal funding, the NIH has been given a right to make the work publicly available in PubMed Central.

- NIH grants P01 HL114501 (to JAE), R01 HL155558 (to CGL), and T32 HL134625 (to TS).
- Department of Defense grant W81XWH-22-1-0041 (to CGL).
- National Research Foundation (NRF) grant RS-2024-00405542 funded by the Korea government (MSIT) (to SJS and CGL).

## Supplementary Material

Supplemental data

Supporting data values

## Version 1 04/28/2026

In-Press Preview

## Version 2 07/08/2026

Electronic publication
