# Supplementary material for: Bispecific targeting of CHI3L1 and PD-1 as a therapeutic strategy for pulmonary fibrosis
Source: JCI Insight. 2026 Apr 28;11(13):e201609. doi: 10.1172/jci.insight.201609 (PMC13460889; doi:10.1172/jci.insight.201609)
Supplement: Supplementary file 1 [file jciinsight-11-201609-s181.pdf]

Supplemental Information

Supplemental Figure 1-9

Supplemental Table 1. Antibodies and reagents used in this study.

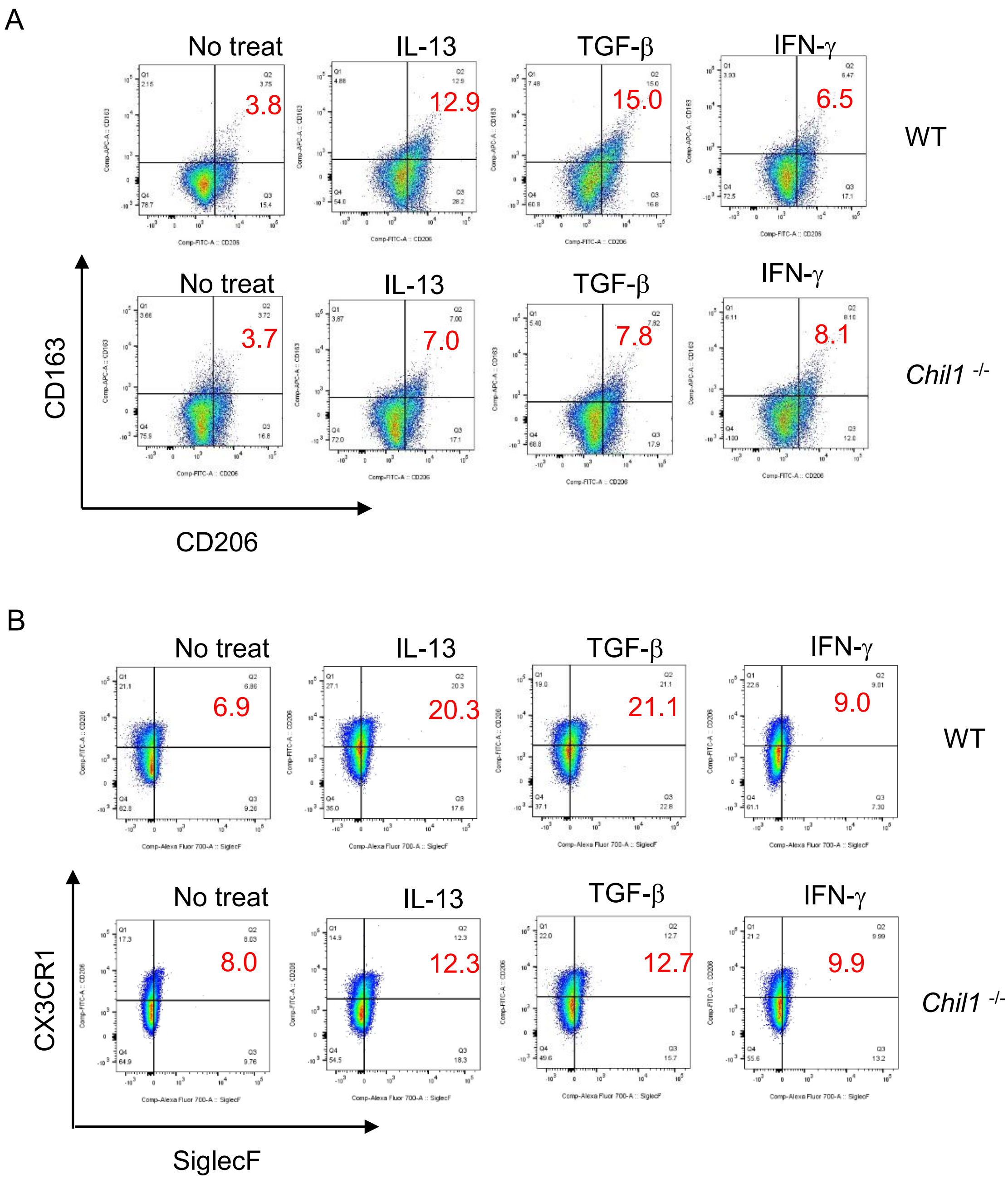

Supplementary Figure 1. CHI3L1 promotes M2 macrophage polarization in response to IL-13 or TGF-β. Bone marrow-derived macrophages (BMDMs) were isolated from wild-type (WT) and CHI3L1-null (*Chil1*$^{-/-}$) mice and stimulated with recombinant IL-13 (20 ng/mL), TGF-β_1 (5 ng/mL), and IFN-γ (20 ng/mL) separately for 72 hours. Cells were then analyzed by flow cytometry. (A) Frequency of CD206$^+$/CD163$^+$ M2 macrophages. (B) Frequency of CX3CR1$^+$/SiglecF$^+$ profibrotic macrophages.

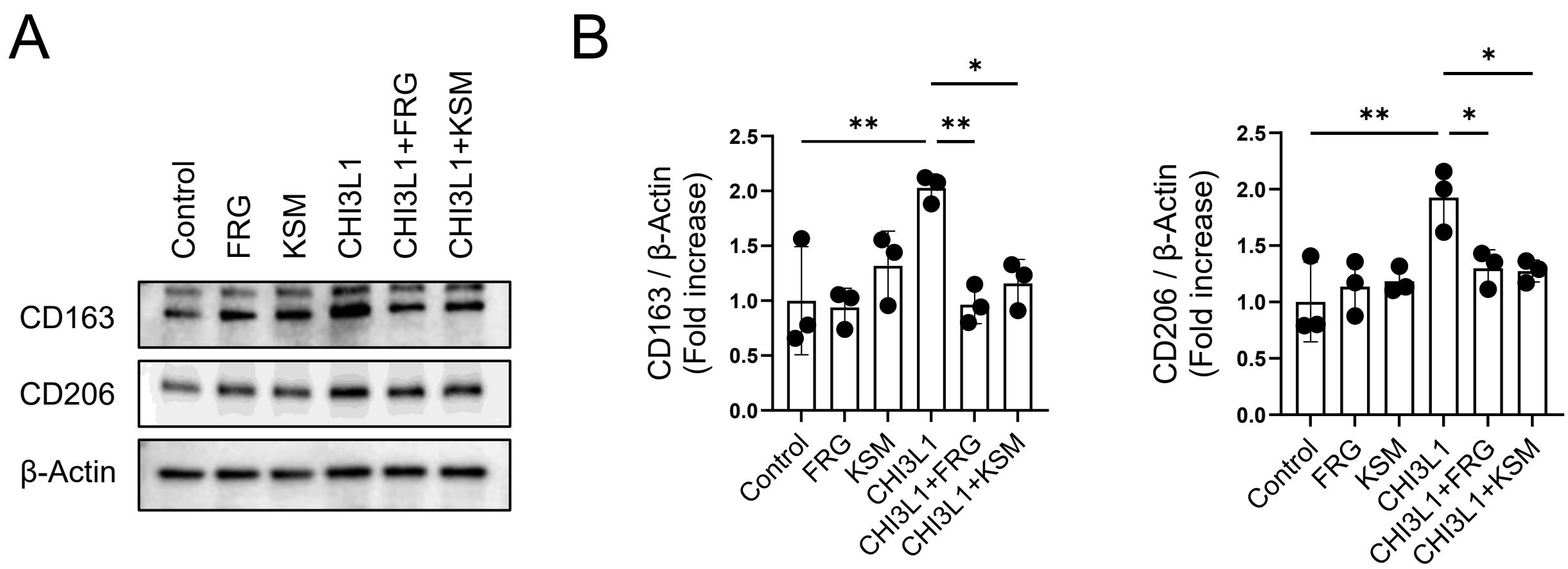

Supplementary Figure 2. CHI3L1 drives M2 polarization of murine bone marrow-derived macrophages (BMDM). Mouse bone-marrow-derived macrophages were stimulated with recombinant CHI3L1 (500 ng/mL) with or without anti-CHI3L1 antibody (FRG, 250 ng/mL) or kasugamycin (KSM, 250 ng/mL) for 24 hours. Expression of CD163 and CD206 was then evaluated by Western blot analysis of CD163 and CD206. β-actin was used as a loading control. (A) Representative Western gel photograph. (B) The bar graphs in panel B represent densitometric quantification of Western blot signals; values in panels A-C represent the mean ± SEM. **p* < 0.05, ***p* < 0.01 by one-way ANOVA with multiple comparisons.

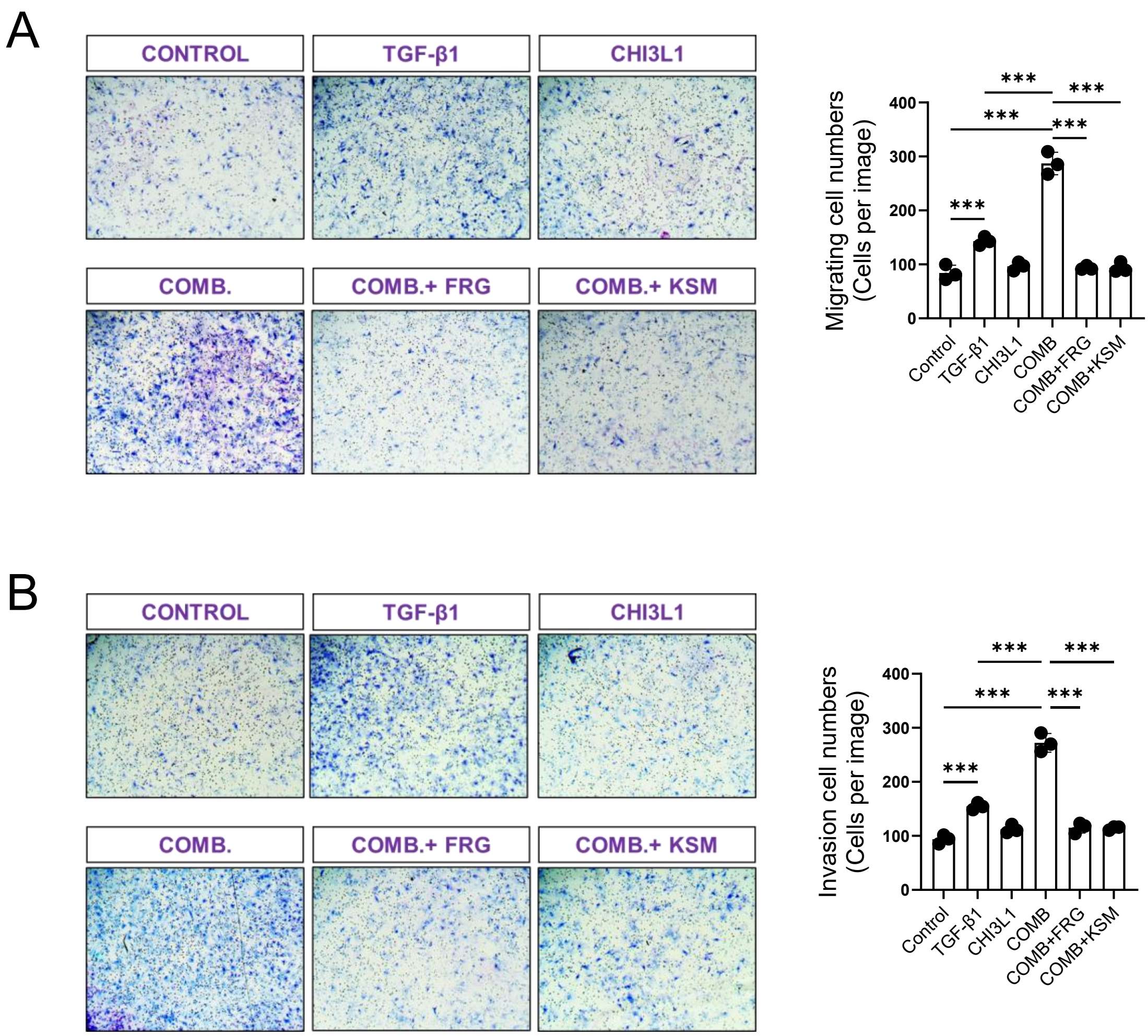

Supplemental Figure 3. CHI3L1 enhances TGF-β_1-induced migration and invasion of murine lung fibroblasts. Murine lung-derived fibroblasts were treated with recombinant CHI3L1 (500 ng/mL) and/or TGF-β_1 (5 ng/mL) with or without FRG (250 ng/mL) or KSM (250 ng/mL). (A-B) Representative images of fibroblast migration (A) and invasion (B) assays. The number of cells that migrated or invaded through the membrane was counted. The values of bar graphs in the right panels of each assay represent mean ± SEM from 3 independent experiments. ***$p < 0.001$ by one-way ANOVA with multiple comparisons. COMB, combined treatment with CHI3L1 and TGF-β_1.

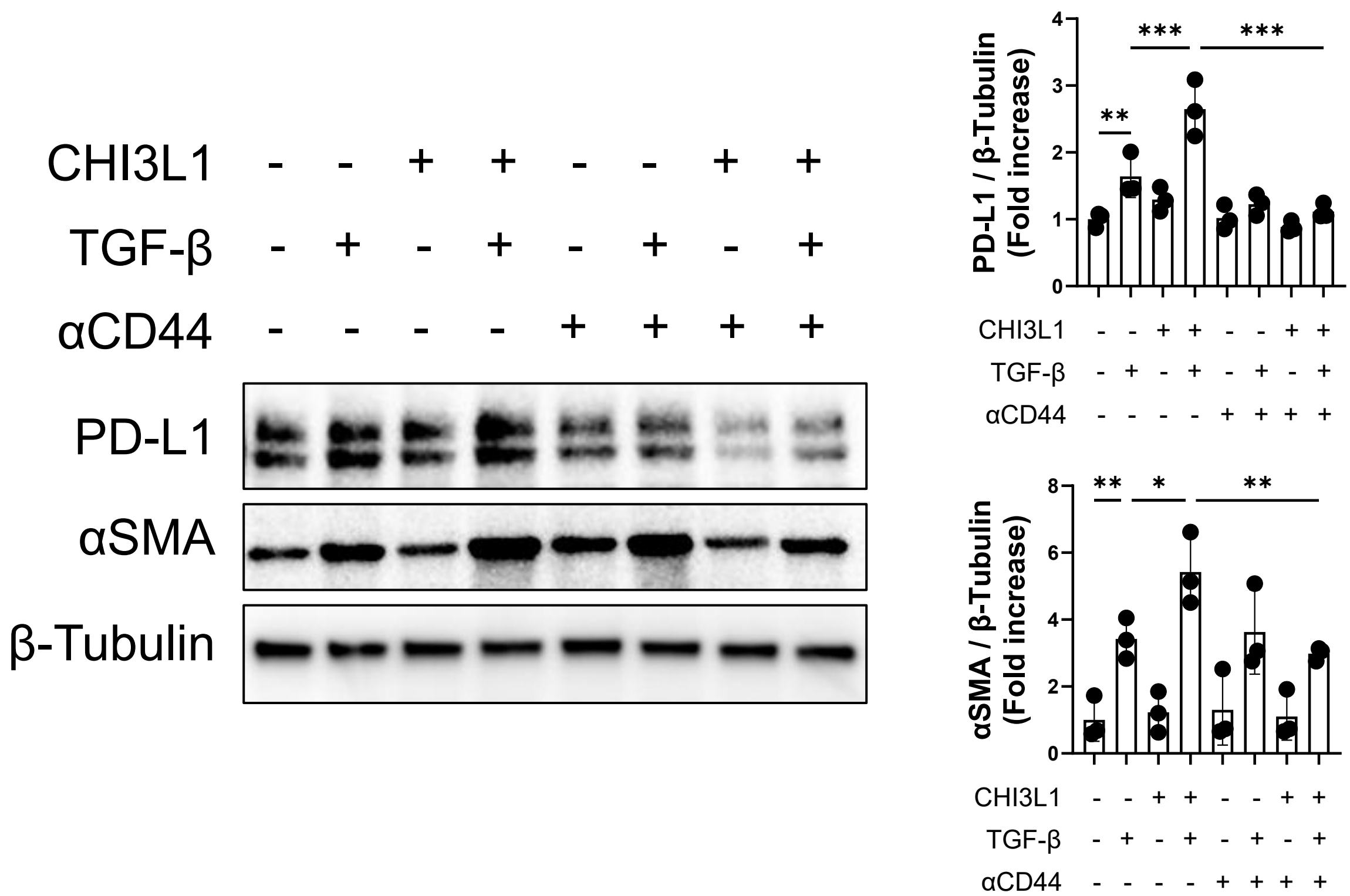

Supplementary Figure 4. Role of CD44 in CHI3L1/TGF-β stimulated expression of α-SMA and PD-L1 expression in NHLFs. Western blot analysis of PD-L1 and α-SMA expression in lung fibroblasts stimulated with recombinant CHI3L1 (500 ng/mL) and/or TGF-β_1 (5 ng/mL) in the presence or absence of neutralizing anti-CD44 antibody (Thermo Fisher Scientific, 5 μg/mL). Bar graphs represent densitometric quantification of protein expression (mean ± SEM). *$p < 0.05$, **$p < 0.01$, ***$p < 0.001$ by one-way ANOVA with multiple comparisons.

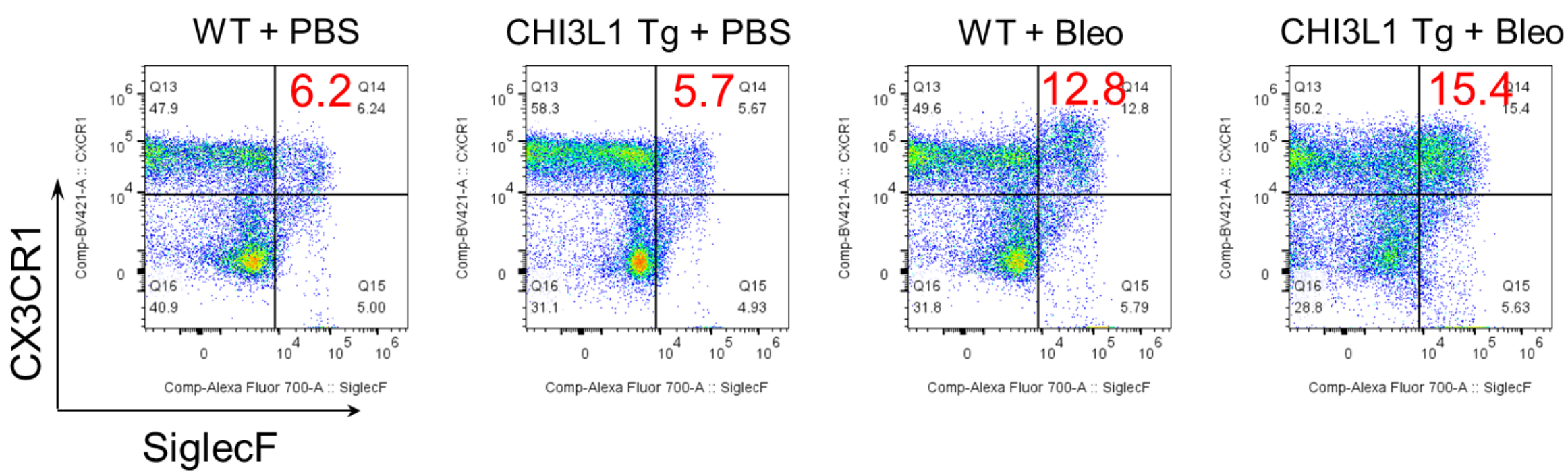

Supplementary Figure 5. CHI3L1 increases profibrotic macrophage activation in bleomycin-challenged lungs. Six to eight weeks old WT and CHI3L1 Tg mice were challenged with PBS or bleomycin, then the mice were sacrificed 14 days after bleomycin challenge and subjected to analysis. (A) Flow cytometric evaluations on CX3CR1^{+} and SiglecF^{+} macrophages in lungs of WT and CHI3L1 transgenic (Tg) mice.

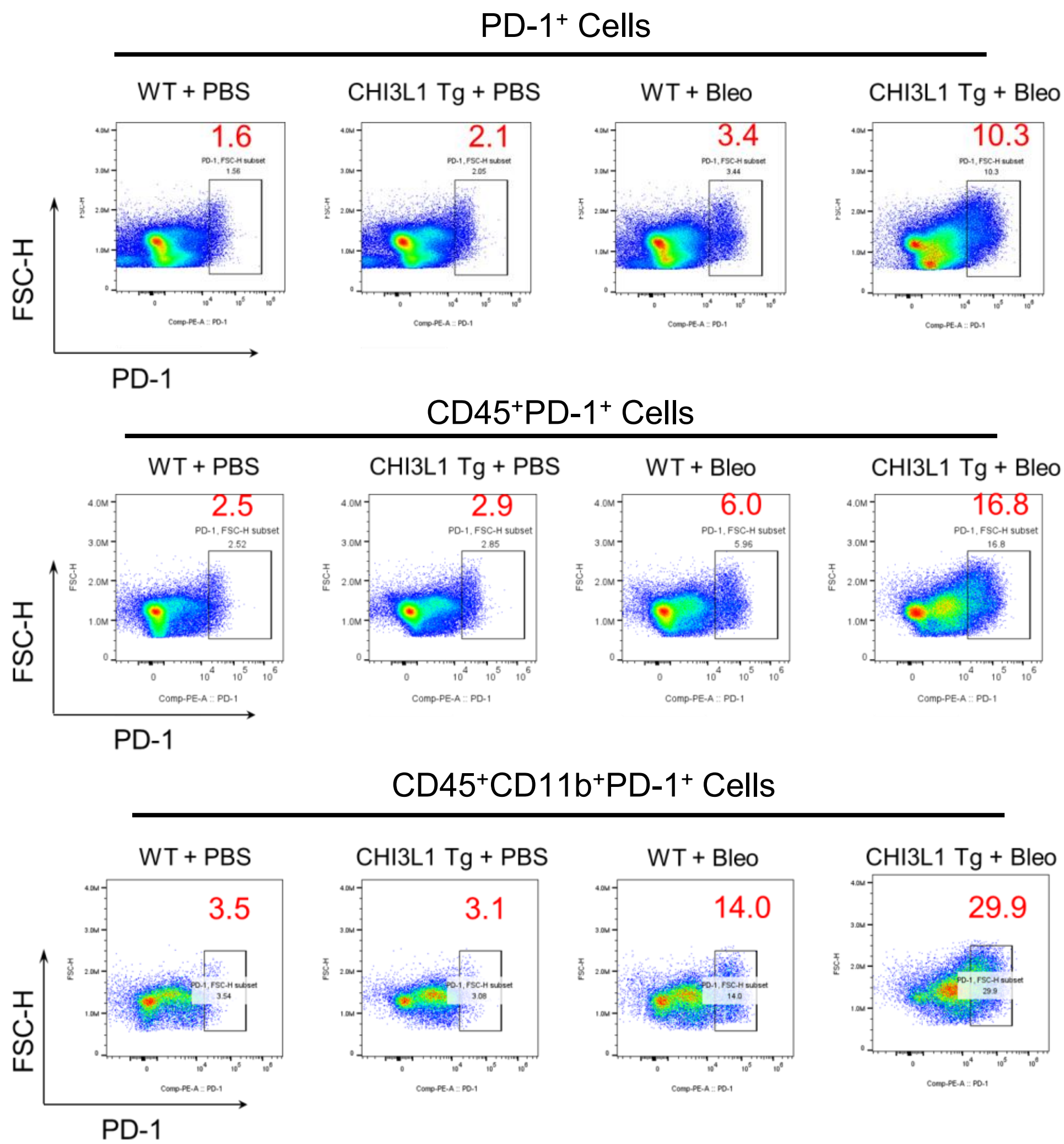

Supplementary Figure 6. CHI3L1 increases total and PD-1–expressing inflammatory cells in bleomycin-challenged lungs. Six to eight weeks old WT and CHI3L1 Tg mice were challenged with PBS or bleomycin, then the mice were sacrificed 14 days later for analysis. Flow cytometric analysis demonstrated increased numbers of total inflammatory cells and $CD45^+/PD\text{-}1^+$ cells, including $CD45^+/CD11b^+/PD\text{-}1^+$ macrophages, in the lungs of CHI3L1 Tg mice compared with WT controls.

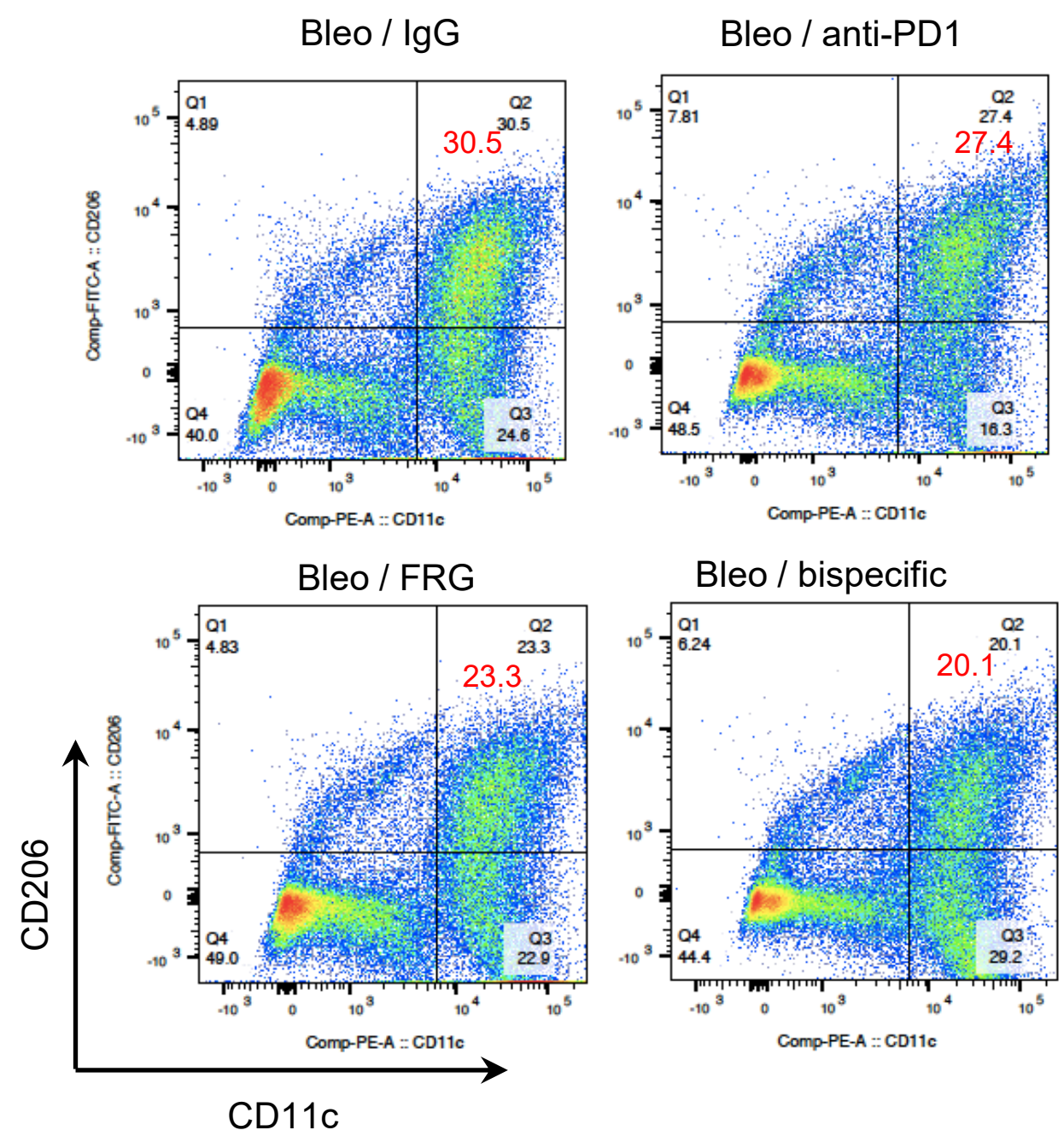

Supplementary Figure 7. Regulation of profibrotic macrophage activation in bleomycin-challenged lungs by mono- and bispecific anti-CHI3L1 antibodies. FACS analysis of $CD45^+/F4/80^+/CD11c^+/CD206^+$ profibrotic macrophages.

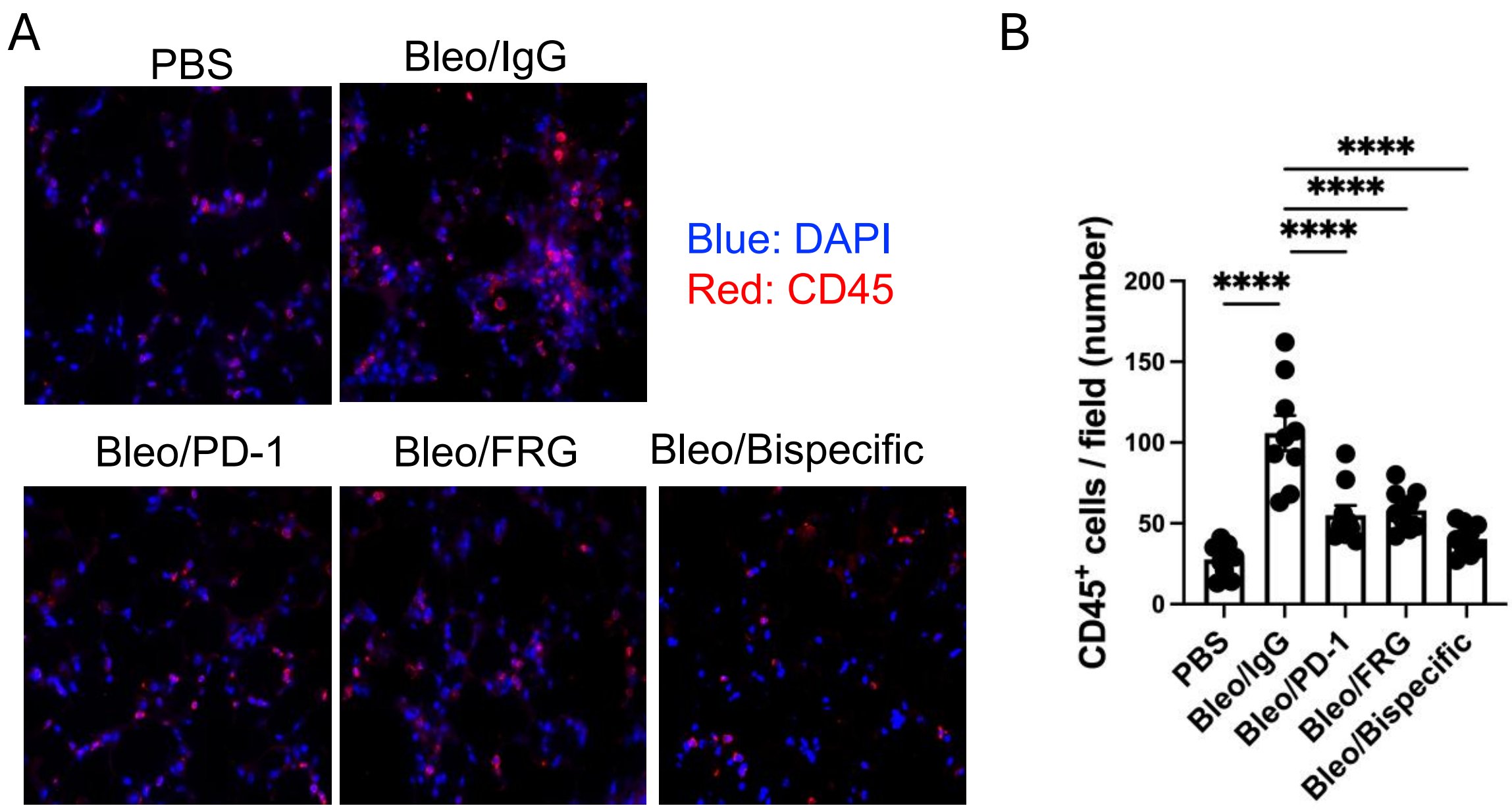

Supplementary Figure 8. Evaluation of lung tissue inflammation following antibody treatment. Lung tissue inflammation was assessed by immunostaining for $CD45^+$ leukocytes. The number of infiltrating $CD45^+$ cells (red) in lung sections from control mice and mice treated with anti-CHI3L1 (FRG), anti-PD-1, or bispecific antibodies was quantified. (A) Representative images of lung sections stained with a fluorescent anti-CD45 antibody. (B) Quantification of $CD45^+$ cells per high-magnification field (×40).

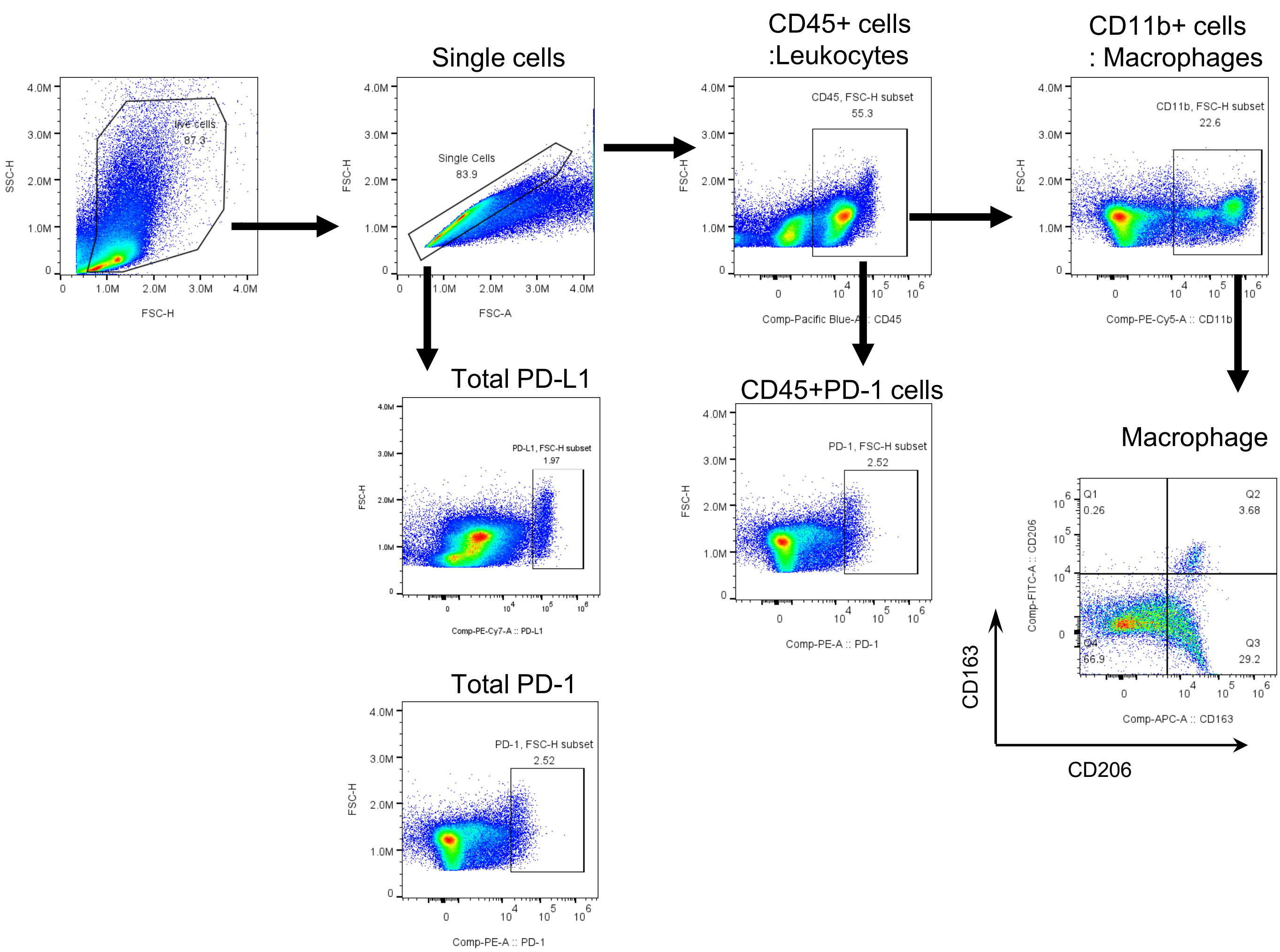

Supplementary Figure 9. Gating strategies used for flow cytometry in this study.

Supplemental Table 1 – Detailed material information

Cells and Mice	Source	Catalog Number or Identifier
THP-1	ATCC	Cat#: TIB-202; RRID: CVCL_0006
Normal Human Lung Fibroblasts (NHLF)	ATCC	Cat#: PCS-201-013
Mouse: C57BL/6J (WT)	The Jackson Laboratory	RRID:IMSR_JAX:000664
Mouse: *Chil1*$^{-}$/$^{-}$ (C57BL/6J Background)	This paper; previously described in (20, 27).	N/A
Mouse: CHI3L1 (YKL) transgenic (C57BL/6J Background)	This paper; previously described in (20, 27).	N/A

Antibodies	Source	Catalog Number or Identifier
APC anti-mouse CD163 Monoclonal Antibody (TNKUPJ) (Diluted at 1:100 for FACS)	Thermo Fisher Scientific	Cat#: 17-1631-82; RRID: AB_2784646
FITC anti-mouse CD206 Monoclonal Antibody (MR5D3) (Diluted at 1:100 for FACS)	Thermo Fisher Scientific	Cat#: MA5-16870; RRID: AB_2538348
Brilliant Violet 421™ anti-mouse CX3CR1 Antibody (SA011F11) (Diluted at 1:100 for FACS)	BioLegend	Cat#: 149023; RRID: AB_2565706
Alexa Fluor™ 700 anti-mouse CD170 (Siglec F) Monoclonal Antibody (1RNM44N) (Diluted at 1:100 for FACS)	Thermo Fisher Scientific	Cat#: 56-1702-82; RRID: AB_2637127
PE anti-mouse CD279 (PD-1) Monoclonal Antibody (J43) (Diluted at 1:50 for FACS)	Thermo Fisher Scientific	Cat#: 12-9985-82; RRID: AB_466295
PE-Cyanine7 anti-mouse CD274 (PD-L1, B7-H1) Monoclonal Antibody (MIH5) (Diluted at 1:100 for FACS)	Thermo Fisher Scientific	Cat#: 25-5982-82; RRID: AB_2573509
Pacific Blue™ anti-mouse CD45 Antibody (30-F11) (Diluted at 1:100 for FACS)	BioLegend	Cat#: 103126; RRID: AB_493535
PE/Cyanine5 anti-mouse/human CD11b Antibody (RUO) (Diluted at 1:100 for FACS)	BioLegend	Cat#: 101210; RRID: AB_312793
PE anti-human CD163 Monoclonal Antibody (GHI/61)	Thermo Fisher Scientific	Cat#: A15792; RRID: AB_2534

(Diluted at 1:100 for FACS)		570
APC/Cyanine7 anti-human CD 206 (MMR) Antibody (15-2) (Diluted at 1:100 for FACS)	BioLegend	Cat#: 321120; RRID: AB_2144930
Phospho-SMAD2 (Ser465/467) Rabbit mAb (138D4) (Diluted at 1:1000 for WB)	Cell Signaling Technology	Cat#:3108; RRID: AB_490941
Phospho-p44/42 MAPK (Erk1/2) (Thr202/Tyr204) Antibody (Polyclonal) (Diluted at 1:1000 for WB)	Cell Signaling Technology	Cat#: 9101; RRID: AB_331646
Phospho-Akt (Ser473) Rabbit mAb (193H12) (Diluted at 1:1000 for WB)	Cell Signaling Technology	Cat#:4058; RRID: AB_331168
SMAD2 Mouse mAb (L16D3) (Diluted at 1:1000 for WB)	Cell Signaling Technology	Cat#: 3103; RRID: AB_490816
p44/42 MAPK (Erk1/2) Antibody (Polyclonal) (Diluted at 1:1000 for WB)	Cell Signaling Technology	Cat#: 9102; RRID: AB_330744
Akt (pan) Rabbit mAb (11E7) (Diluted at 1:1000 for WB)	Cell Signaling Technology	Cat#: 4685; RRID: AB_2225340
Anti-alpha smooth muscle Actin antibody (EPR5368) (Diluted at 1:10000 for WB)(Diluted at 1:200 for ICC)	Abcam	Cat#: ab124964; RRID: AB_11129103
CD44 XP® Rabbit mAb (E7K2Y) (Diluted at 1:1000 for WB)	Cell Signaling Technology	Cat#: 37259; RRID: AB_2750879
CD163 anti-human Polyclonal Antibody (Diluted at 1:1000 for WB)	Thermo Fisher Scientific	Cat#: PA5-109327; RRID: AB_2854738
CD206 (MMR) Recombinant Rabbit Monoclonal Antibody (JF0953) (Diluted at 1:1000 for WB)	Thermo Fisher Scientific	Cat#: MA5-32498; RRID: AB_2809775
PD-L1 XP® Rabbit mAb (E1L3N®) (Diluted at 1:1000 for WB)	Cell Signaling Technology	Cat#: 13684; RRID: AB_2687655
beta Actin Antibody (C4) (Diluted at 1:20000 for WB)	Santa Cruz Biotechnology	Cat#: sc-47778; RRID: AB_626632
β-Tubulin Antibody (Polyclonal) (Diluted at 1:1000 for WB)	Cell Signaling Technology	Cat#: 2146; RRID: AB_2210545
Mouse PD-L1 Rabbit mAb(D4H1Z) (Diluted at 1:1000 for WB)	Cell Signaling Technology	Cat#: 60475; RRID: AB_2924680
Mouse MMR/CD206 Antibody (Polyclonal)(Diluted at 1:200 for IHC)	R&D Systems	Cat#: AF2535; RRID: AB_2063012
Mouse CD163 antibody (EPR19518) (Diluted at 1:200 for IHC)	Abcam	Cat#: ab182422; RRID: AB_2753196

PD-L1 Polyclonal Antibody (Diluted at 1:100 for IHC)	Thermo Fisher Scientific	Cat#: PA5-20343; RRID: AB_11153819
CD140a (PDGFRA) Monoclonal Antibody (APA5) (Diluted at 1:100 for IHC)	Thermo Fisher Scientific	Cat#: 14-1401-82; RRID: AB_467491
CD44 Monoclonal Antibody (IM7) (treat 2.5 μg/mL for Neutralization)	Thermo Fisher Scientific	Cat#: 14-0441-85; RRID: AB_467247
Purified anti-mouse CD16/32 Antibody (RUO)(Diluted at 1:300 for FACS)	BioLegend	Cat#: 101302; RRID: AB_312801
Anti-Hu Fc Receptor Binding Inhibitor Polyclonal Antibody (Diluted at 1:300 for FACS)	Thermo Fisher Scientific	Cat#: 14-9161-73; RRID: AB_468582
HRP-linked anti-mouse IgG (Diluted at 1:2000 for WB)	Cell Signaling Technology	Cat#: 7076; RRID: AB_330924
Anti-human CHI3L1 antibody (FRG) (Neutralization)	This paper; previously described in (21).	N/A
Anti-mouse CHI3L1 antibody (FRG) (*In vivo*)	This paper; previously described in (21).	N/A
Anti-CHI3L1×PD-1 antibody (*In vivo*)	This paper; previously described in (21).	N/A
Anti-PD-1 antibody (*In vivo*)	This paper; previously described in (21).	N/A
CD163 (E2H3C) Rabbit Monoclonal Antibody (Diluted at 1:1,000 for mouse WB)	Cell Signaling Technology	Cat#: 68922; RRID: AB_3105898
CD45 Monoclonal Antibody (30-F11)(Diluted at 1:100 for IHC)	Thermo Fisher Scientific	Cat#: 14-0451-82; RRID: AB_467251
CD206/MRC1 (E6T5J) Rabbit Monoclonal Antibody (Diluted at 1:1,000 for mouse WB)	Cell Signaling Technology	Cat#: 24595; RRID: AB_2892682

**Oligonucleotides**	**Source**	**Catalog Number or Identifier**
PCR Primers	This study	"Methods"
AccuTarget™ Negative Control siRNA	Bioneer	Cat#: SN-1003
AccuTarget™ siRNA targeting human PD-L1#1	Bioneer	Cat#: 29126-1
AccuTarget™ siRNA targeting human PD-L1#2	Bioneer	Cat#: 29126-2
AccuTarget™ siRNA targeting mouse CD44#1	Bioneer	Cat#: 12505-1
AccuTarget™	Bioneer	Cat#: 12505-2

siRNA targeting mouse CD44# 2		

Chemicals and other re agents	Source	Catalog Number or Identifier
PMA (Phorbol 12-myristate 13-acetate)	Merck	Cat#: P1585
Kasugamycin hydrochloride	Merck	Cat#: K4013
Doxycycline hyclate	Merck	Cat#: D9891
Trypsin-EDTA Solution, 1X	ATCC	Cat#: 30-2101
RIPA lysis buffer	Thermo Fisher Scientific	Cat#: 89901
1X RBC Lysis Buffer	Thermo Fisher Scientific	Cat#: 00-4333-57
SDS Laemmli buffer	Boston BioProducts	Cat#: BP-111R
SuperSignal™ West Pico PLUS Chemiluminescent Substrate	Thermo Fisher Scientific	Cat#: 34580
SuperSignal™ West Femto Max imum Sensitivity Substrate	Thermo Fisher Scientific	Cat#: 34096
10x Citrate Buffer pH 6.0	Abcam	Cat#: ab64214
Protein Block, Serum-Free	Agilent/Dako	Cat#: X090930-2
DMEM medium	Gibco	Cat#: 11965084
RPMI 1640 medium	Gibco	Cat#: 11875085
FGM®-2 Fibroblast Growth Medium-2 BulletKit®	Lonza	Cat#: CC-3132
Fetal Bovine Serum	Gibco	Cat#: A5256501
PBS, pH 7.4	Gibco	Cat#: 10010023
Penicillin-Streptomycin-Glutamine (100X)	Gibco	Cat#: 10378016
VECTASHIELD® Antifade Mou nting Medium with DAPI	Vector laboratories	Cat#: H-1200-10
Bleomycin	Hikma Pharmaceuticals	Cat#: NDC 0143-9241-0 1
Recombinant human CHI3L1	R&D Systems	Cat#: 2599-CH
Recombinant human TGF-β1	R&D Systems	Cat#: 11409-BH
Recombinant human IFN-γ	R&D Systems	Cat#: 285-IF
Recombinant human PDGF-BB	Peprotech	Cat#: 100-14B
Recombinant mouse IL-13	R&D Systems	Cat#: 413-ML
Recombinant mouse TGF-β1	R&D Systems	Cat#: 240-B
Recombinant mouse M-CSF	R&D Systems	Cat#: 416-ML
BioCoat™ Matrigel Invasion Ch ambers (8 μm)	Corning	Cat#: 354480
Transwell Inserts, 24-well, 8 μ m	Corning	Cat#: 353097
QIAzol Lysis Reagent	QIAGEN	Cat#: 79306
RNeasy Mini Kit	QIAGEN	Cat#: 74106

iScript™ cDNA Synthesis Kit	Bio-Rad	Cat#: 1708891
Lipofectamine RNAiMAX	Thermo Fisher Scientific	Cat#: 13778150
Kwik-Diff™ Stain Kit	Thermo Fisher Scientific	Cat#: 9990701
Lung Dissociation Kit, mouse	Miltenyi Biotec	Cat#: 130-095-927

**Software**	**Source**	**Catalog Number or Identifier**
FlowJo (v10.10)	BD Bioscience	RRID: SCR_008520
GraphPad Prism (v10)	GraphPad	RRID: SCR_002798
R (v4.5.1)	R Foundation	RRID: SCR_001905
ggplot2	CRAN	cran.r-project.org/package=ggplot2
Python (v3.10)	Python Software Foundation	https://www.python.org
Scanpy (v1.9.8)	Wolf et al., 2018 (Genome Biology)	RRID:SCR_018139; https://scanpy.readthedocs.io
SciPy (v1.11.4)	Virtanen et al., 2020 (Nat Methods)	RRID:SCR_008058; https://scipy.org
seaborn (v0.13.2)	Waskom et al., 2021	RRID:SCR_018132; https://seaborn.pydata.org
ImageJ (v1.54)	NIH	RRID:SCR_002285

**Other**	**Source**	**Catalog Number or Identifier**
BD FACSAria™ III Cell Sorter	BD Bioscience	
Cytek® Aurora	Cytek® Biosciences	
